# Biochemical and proteomic response of the freshwater green alga *Pseudochlorella pringsheimii* to iron and salinity stressors

**DOI:** 10.1186/s12870-023-04688-9

**Published:** 2024-01-10

**Authors:** Mostafa M. S. Ismaiel, Michele D. Piercey-Normore, Christof Rampitsch

**Affiliations:** 1 Department of Botany and Microbiology, Faculty of Science, Zagazig University, Zagazig, 44519 Egypt; 2 Faculty of Science, Algoma University, Sault Ste Marie, ON P6A 2G4 Canada; 3 Morden Research and Development Centre, Agriculture and Agri-Food Canada, Morden, MB R6M 1Y5 Canada

**Keywords:** Antioxidants, ATP, Carbohydrate, Metabolism, Microalgae, Stress

## Abstract

**Background:**

*Pseudochlorella pringsheimii* (*Ppr)* is a green unicellular alga rich with chlorophyll, carotenoids, and antioxidants. As a widespread organism, *Ppr* must face, and adapt to, many environmental stresses and these are becoming more frequent and more extreme under the conditions of climate change. We therefore focused on salinity induced by NaCl and iron (Fe) variation stresses, which are commonly encountered by algae in their natural environment.

**Results:**

The relatively low stress levels improved the biomass, growth rate, and biochemical components of *Ppr*. In addition, the radical-scavenging activity, reducing power, and chelating activity were stimulated by lower iron concentrations and all NaCl concentrations. We believe that the alga has adapted to the stressors by increasing certain biomolecules such as carotenoids, phenolics, proteins, and carbohydrates. These act as antioxidants and osmoregulators to protect cell membranes and other cellular components from the harmful effects of ions. We have used SDS-PAGE and 2D-PAGE in combination with tandem mass spectrometry to identify responsive proteins in the proteomes of stressed vs. non-stressed *Ppr*. The results of 2D-PAGE analysis showed a total of 67 differentially expressed proteins, and SDS-PAGE identified 559 peptides corresponding to 77 proteins. Of these, 15, 8, and 17 peptides were uniquely identified only under the control, iron, and salinity treatments, respectively. The peptides were classified into 12 functional categories: energy metabolism (the most notable proteins), carbohydrate metabolism, regulation, photosynthesis, protein synthesis, stress proteins, oxido-reductase proteins, transfer proteins, ribonucleic-associated proteins, hypothetical proteins, and unknown proteins. The number of identified peptides was higher under salinity stress compared to iron stress.

**Conclusions:**

A proposed mechanism for the adaptation of *Ppr* to stress is discussed based on the collected data. This data could serve as reference material for algal proteomics and the mechanisms involved in mediating stress tolerance.

**Supplementary Information:**

The online version contains supplementary material available at 10.1186/s12870-023-04688-9.

## Background

Recent climate change and anthropogenic activities have led to increase extreme weather events and higher levels of hazardous materials and pollution in the natural environment. The widespread distribution of algae in many environments inevitably results in their exposure to pollution and various stresses and, in many cases, they represent the first organisms in the food chain exposed to environmental stresses [1–3].

Previous literature has classified the stress that algal cells experience and has sought to understand the mechanisms by which can endure and withstand stress. Researches on stressed algae have uncovered potential new functions for algae in the remediation and purification of polluted water, as well as in the production of resilient varieties with high economic value [4–8].

Heavy metals (e.g. iron) and salinity stressors have attracted much attention because of their widespread presence in the environment and their substantial impact on the survival, secondary metabolites, and food chains of various organisms [6, 9, 10]. Iron is a crucial microelement for the photosynthesis and metabolic processes of algae. However, excessive concentrations of iron can lead to stress or toxicity in algal growth, biochemical components, and metabolic processes [11–13]. The high concentration of iron may be attributed to the accumulation of salts leaching from upland slopes, waterlogged soils, or other types of soil into nearby regions. Additionally, the discharge of industrial waste and domestic wastewater may pose serious problems for the nearby ecosystem [14]. The toxicity of iron may be related to its involvement in the Fenton or Haber-Weiss reaction, which generates reactive oxygen species (ROS) such as superoxide and hydroxyl radicals that cause oxidative stress in living cells [12, 13].

Salinization of soils and waters is a global issue that has greatly increased due to drought, temperature fluctuations, irrigation, and pollution [2, 15]. The toxic effect of salinity can be attributed to a disturbance of cellular homeostasis due to an imbalance of ions, osmotic fluctuations, and the production of ROS [16–19].

Microalgae are known to modify their cells to respond effectively to stress [20, 21]. The severity of the stress can directly impact the growth and biomass of the microalgae. Additionally, various biochemical components such as chlorophyll, carotenoids, proteins, carbohydrates, and other protective molecules can be modulated to act as antioxidants and stress response mechanisms [1, 4, 5, 18, 22].

Numerous studies have been conducted to illustrate the impact of iron on the growth, biomass, pigments, lipid accumulation, and fatty acid profile of algal species such as *Scenedesmus dimorphus* [23], *Tetradesmus obliquus* [12], *Chlorella sorokiniana* [11]. The effect of salinity has also been observed in *Acutodesmus obliquus, Chlorella vulgaris* [10, 22], *Scenedesmus* sp. [16]. However, further research on the protein profile following stress exposure and the identification of the mechanisms involved are necessary for a comprehensive understanding of the acclimation responses of algal species under stress.

Proteomics is a valuable tool for studying the potential changes in gene expression in a living organism under stress. Most of the proteins modulated under stress are related to photosynthesis, regulation, carbohydrate, and energy metabolism, antioxidants, stress proteins such as heat shock proteins, etc. [6, 18, 24]. However, there are limited studies on microalgae under stress, with some examples including the model diatom *Phaeodactylum tricornutum* [25], and the cyanobacterium *Arthrospira* (*Spirulina*) *platensis* [18] under iron stress. Other examples include the green algae *Dunaliella salina* [26–28], and *Chlamydomonas reinhardtii* [24, 29] under salinity stress. Many other species have not been studied in this context.

*Pseudochlorella pringsheimii* (*Ppr*) is a widely distributed unicellular eukaryotic chlorophyte (green microalga). While there have been studies on its growth, biochemical constituents, and antioxidants under Cd, Pb, and nitrogen stresses [7, 30], the effects of iron and salinity stress on this species have not been thoroughly examined. Therefore, this study aimed to investigate the impact of iron and salinity stress on *Ppr* in order to understand the potential adaptive mechanisms employed by the green alga in terms of growth, pigment, biochemical contents, and antioxidant activity. Moreover, the proteome of *Ppr* was analyzed under both normal and stress conditions to gain insight into how green algae respond differently to stresses (on the proteomic level).

## Materials and methods

### Alga, culture, and stressors

*Pseudochlorella pringsheimii* MIYA 102 (*Ppr*) (Trebouxiophyceae, Chlorophyta), a green freshwater eukaryotic alga, was obtained from the Phycology Lab, Faculty of Science, Zagazig University, Egypt. The alga was maintained on Bold’s Basal medium (designed for freshwater algae), as described by Bischoff and Bold [31]. Culture media (49 mL) were dispensed in 125-mL Erlenmeyer flasks. Two stressors were applied: iron (0.025, 0.045, 0.09, 0.18, 0.35, and 0.7 mM FeSO_4_.7H_2_O) and salinity (8.5, 17, 34, 68, 100, and 136 mM NaCl) based on a preliminary experiment (unpublished observations), where the concentration beyond that range was highly (> 95%) lethal to obtain a proper biomass for the analysis of the selected algal components. The original standard concentrations of Fe and NaCl in Bold’s Basal medium were 0.018 mM and 0.43 mM, respectively. The flasks were cotton plugged and sterilized by autoclaving at 121 °C for 20 min. A 1 mL-sized inoculum (5 × 10^5^ cells.mL^− 1^ of algal mid-log stock culture) was used to inoculate the cooled culture flasks. The flasks were incubated at 30 ± 0.5 °C (the optimum temperature for this alga, data not shown) and illuminated with continuous fluorescent light at 90 µmol photons m^− 2^ s^− 1^ for 10 days (the middle of the log-growth phase). The algal cells were scattered by hand shaking twice daily.

The algal cells were harvested on the 10^th^ day of incubation by centrifugation at 6,000 *xg* for 10 min at 4 °C using Sorvall Legend X1R centrifuge (Thermo Scientific). The cells were then washed with 10 mM Na_2_-EDTA to remove any adsorbed metals on the cell surface, followed by a wash with distilled water. After washing, the cells were weighed and expressed as fresh weight (g FW), frozen in liquid nitrogen, and stored at − 80 °C until used. The chemicals used were of fine analytical grade, purchased from Sigma-Aldrich, Steinheim, Germany (unless otherwise cited). The analysis experiments were designed to include three biological replicates.

### Growth and pigment analyses

The biomass yield (dry weight; DW) was determined by drying the algal cells in an electric oven at 105 °C until a constant weight was achieved [32]. The specific growth rate (µ) of the alga incubated under stressors was calculated using the equation developed by Yong et al. [33]:

$$\mu = [ln({W_t}/{W_0})/d]$$

where W_t_ and W_0_ are the final and initial mass (DW) and d is the time of incubation (10 days).

The quantitative analysis of chlorophyll a and b (Chl *a* & *b*) was conducted following the method outlined by the American Public Health Association (APHA) [32] using a 85% (v/v) aqueous acetone solution as described by Ismaiel [30]. In brief, 100 mg of fresh algal weight was homogenized in pre-cooled acetone using a mortar, and then centrifuged at 4000 *xg* for 10 min. The supernatant was adjusted to 10 mL with the acetone and the different pigment fractions were calculated spectrophotometrically using the following equations:

$${\rm Chl}\; a = 10.3{A_{663}}-0.918{A_{644}}$$

$${\rm{Chl}}\;b = 19.7{A_{644}}-3.87{A_{663}}$$

$${\rm{Carotenoids }} = 4.2{A_{452.5}}-\left( {0.0264{\rm{ Chl}}\,a + 0.496{\rm{ Chl}}\,b} \right)$$

where Chl *a*, Chl *b*, and Carotenoids are the concentrations of pigment fraction in the acetone extract (mg.L^− 1^), respectively; and *A*_663_, *A*_644_, and *A*_452.5_ are the absorbances (with a 1-cm light path) at the indicated wavelengths.

### Biochemical and antioxidants analyses

#### Extraction

The algal extract for the biochemical analyses was prepared by vortexing algae equivalent to 100 mg of algal dry weight (harvested after the 10^th^ day of the incubation period) with glass beads (0.45–0.5 mm diameter) in 2 mL of sterile distilled water, at 4 °C, following the method described by Ismaiel [30].

#### Protein and soluble carbohydrate

The soluble protein content of the algal sample was measured using a Bio-Rad protein assay kit (BioRad, USA) following the Bradford method [34]. The direct quantification of carbohydrates in the algal extract was labeled as the soluble carbohydrates fraction. Total soluble carbohydrates were extracted using 1 N NaOH in a boiling water bath for 2 h following the procedures of Payne and Stewart [35]. The quantification of carbohydrates fractions was carried out using the phenol-sulfuric acid method [36].

#### Radical scavenging activity

The radical scavenging activity of algal extracts was assessed using the method described by Sánchez-Moreno et al. [37] with slight modifications as outlined by Ismaiel [30]. In summary, 100 µL aliquots of each algal sample extract were added to 15-mL screw-capped tubes containing 3.9 mL of 0.06 mM DPPH (2,2-diphenyl-1-picrylhydrazyl radical) methanol-solution and incubated at ambient temperature for 4 h in continuous shaking (200 rpm) in the dark. The absorbance of the samples was measured as follows:

$${\rm{Radical}}\,{\rm{scavenging}}\,{\rm{activity}}\,\left( \% \right) = ({A_B}-\left( {{A_S} - {A_G}} \right))/{A_B} \times 100$$

Where the absorbance of the DPPH solution alone (blank) is *A*_*B*_; the absorbance of the sample in DPPH solution is *A*_*S*_; the absorbance of the sample in methanol solution alone (without DPPH) is *A*_*G*_; at 515 nm.

#### Reducing power

The reducing power was determined following the method described by Zhu et al. [38]. Two hundred microliter aliquots of the algal extracts were mixed with the assay solution consisted of 0.2 mL of 0.2 M phosphate buffer (pH 6.6) and 0.2 mL of 1% potassium ferricyanide (K_3_Fe (CN)_6_). The assay mixture was incubated at 50 °C for 20 min and allowed to cool. Following this, 0.2 mL of 10% trichloroacetic acid and 0.2 mL of 0.1% FeCl_3_ were added sequently, and the mixture was left for an additional 5 min. The total volume was adjusted to 2 mL with distilled water. The increase in color of the reaction mixture was measured spectrophotometrically at 655 nm indicating the increase in the reducing power of samples. The data was calculated as the change in absorbance (ΔOD) mg^− 1^ DW.

#### Chelating activity

The chelating activity of algal extracts, indicating their ability to chelate ferrous ions, was determined according to Puntel et al. [39]. Two hundred microliter aliquots of the algal extracts were added to a reaction mixture containing 150 µL of 500 µM freshly prepared FeSO_4_ solution and 168 µL of 0.1 M Tris-HCl (pH 7.4) and incubated at ambient temperature for 5 min. Finally, 13 µL of 0.25% 1,10-phenanthroline was added, the total volume was adjusted to 3 mL, and the absorbance was measured at 510 nm. The activity was calculated using the formula:

$${\rm{Chelating}}\,{\rm{activity }}\left( \% \right) = \left( {{A_B}-{A_S}} \right)/{A_B} \times 100$$

where the absorbance of the blank (assay mixture without the algal extract) is *A*_*B*_, and the absorbance of the algal sample is *A*_*S*_ at 510 nm.

The data from the three antioxidant assays were normalized to 100% using the synthetic standard butylated hydroxytoluene (2.5 µg BHT) as the positive control.

### Proteomic analysis

#### Extraction

The algal samples were collected as mentioned above and used to extract total proteins using the acetone/trichloroacetic acid (TCA) precipitation method [40]. Briefly, ≈ 1 g of fresh weight algal samples was homogenized (with an equal volume of 0.45–0.50 mm size glass beads) in glass centrifuge tubes containing 1 mL of pre-chilled acetone, and 10% (w/v) TCA, 0.07% (w/v) dithiothreitol (DTT) and kept at − 20 °C overnight. The tubes were then centrifuged at 10,000 *xg* for 20 min, at − 5 °C. The resulting pellets were washed seven times with pre-chilled acetone/0.07% (w/v) DTT and stored for 30 min at − 20 °C. A stream of nitrogen gas was gently used to dry the algal pellets from any acetone residue, and IEF solution (7 M urea, 2 M thiourea, 4% (w/v) CHAPS, 20 mM DTT, and 0.5% (v/v) of pH 3–10 ampholyte; Bio-Rad Laboratories, Hercules CA, USA) was used to re-dissolve the pellets as described previously [41]. The protein content was quantified using the Bradford assay (BioRad) and adjusted to a concentration of 20 µg (as calculated using BSA as standard) in 20 µL (for 1D SDS-PAGE) and 550 µg (in 450 µL) for the samples of 2-DE.

#### 1D SDS-PAGE

The algal protein extracts were mixed with loading buffer containing 50 mM Tris Hydrochloride (pH 6.8), 100 mM DTT, 2% (w/v) SDS, 0.1% (w/v) bromophenol blue, and 10% glycerol. The mixture was briefly vortexed, centrifuged, and boiled for 2 min. The samples were loaded into a 12% acrylamide gel with a 4% stacking gel as described by Laemmli [42], using a mini gel apparatus (BioRad, USA), and run at 120 V for 15 min. The gel was stained with 1% Coomassie Brilliant Blue R250 for protein visualization.

The entire lanes of control, 0.35 mM Fe, and 136 mM NaCl were sliced up separately into 10 parts of approximately equal size and transferred to 1.5 mL Eppendorf tubes and stored at − 20 °C until further treatment [43]. The gel slices were washed with sterile dH_2_O, NH_4_HCO_3_, acetonitrile, DTT, and Iodoacetamide (IAA) in sequenced steps and concentrations as described by Rampitsch and Bykova [41]. After centrifugation, the supernatant was discarded. Proteins from each gel slice were digested in situ with trypsin (sequencing grade modified trypsin, Promega, Madison, WI, USA) and peptides dissolved in acetonitrile and formic acid in preparation for liquid chromatography–mass spectrometry (LC-MS) analysis.

#### Mass spectrometry and data analysis

Mass spectrometric analysis by LC-ESI–MS/MS was conducted as described by Ismaiel et al. [18]. Briefly, a nanoflow HPLC system (Ultimate 3000: Dionex ThermoFisher, Bremen, Germany) was used to separate peptides using a standard acetonitrile gradient on a C_18_ column (5 μm particle size, 300 Å pores, 10 cm) eluting directly into a linear ion trap mass spectrometer (LTQ XL: Thermo Fisher Scientific, Inc., San Jose CA, USA) at 250 nl.min^− 1^ via nano-electrospray ionization. A 2% v/v ACN (acetonitrile) to 80% v/v ACN gradient in 1% v/v FA (Formic Acid) and 0.5% v/v acetic acid was used for HPLC, with a total run time of 65 min. A “Top 10” programme was used for MS/MS analysis: the ten most abundant peaks in each survey scan were used as precursor ions for an MS^2^ spectrum, with a dynamic exclusion for 30 s.

The obtained mass spectra were analyzed using Mascot MS-MS Ions Search (Matrix Science Ltd., London) and Sonar MS/MS (Proteometrics Ltd., New York) search engines using the nonredundant NCBI protein database. The following parameters were included with each search: trypsin with one missed cleavage permitted; fixed modification of C (cam), variable modifications of M (ox) and NQ (deam); the precursor tolerance was ± 1 Da, MS/MS tolerance was ± 0.8 Da. Automatic decoy searches were included with each query. Proteins were considered correctly identified if more than 2 peptides matched with significant Mascot ions scores (*p* < 0.05 of random assignment).

#### 2D-PAGE

The two-dimensional gel electrophoresis was carried out as described by Rampitsch and Bykova [41]. Briefly, samples were used to rehydrate 24-cm IEFstrips (IPG: GE Healthcare, Mississauga, ON, Canada), pH 4–7, in a Teflon re-swelling tray at ambient temperature covered by a layer of mineral oil to prevent evaporation. Sample strips were focused by a total power of 58 kVh (Multiphor II: GE Healthcare). After focusing, the strips were reduced, alkylated, and equilibrated as recommended by the manufacturer procedures, and separated by12% SDS-PAGE (sodium dodecylsulfate-polyacrylamide gel electrophoresis) gels (via Ettan Dalt-6: GE Healthcare) at a constant power setting of 2.5 W per gel, 30 min, followed by 100 W till the end. The total run time was 4.75 h per 6 gels and 3.5 h per 3 gels. The gels were fixed by 12.5% w/v TCA for 30 min, stained overnight with ≈ 20 mL (per gel) of 1% w/v CBB R-250 (in 95% ethanol), destained by distilled water, and scanned (Epson Expression 1680, Epson, CA, USA) to give TIF images and printed for visual analysis.

### Statistical analysis

Observed data (mean ± standard deviation; SD) were statistically analyzed using SPSS Inc. software version 10.0 (SPSS, Richmond, VA, USA). The significance of the difference among the means was analyzed using a One-way analysis of variance (ANOVA) with Duncan’s multiple range tests at *P* < 0.05.

## Results and discussion

### Biomass and growth rate

**Fig. 1:**
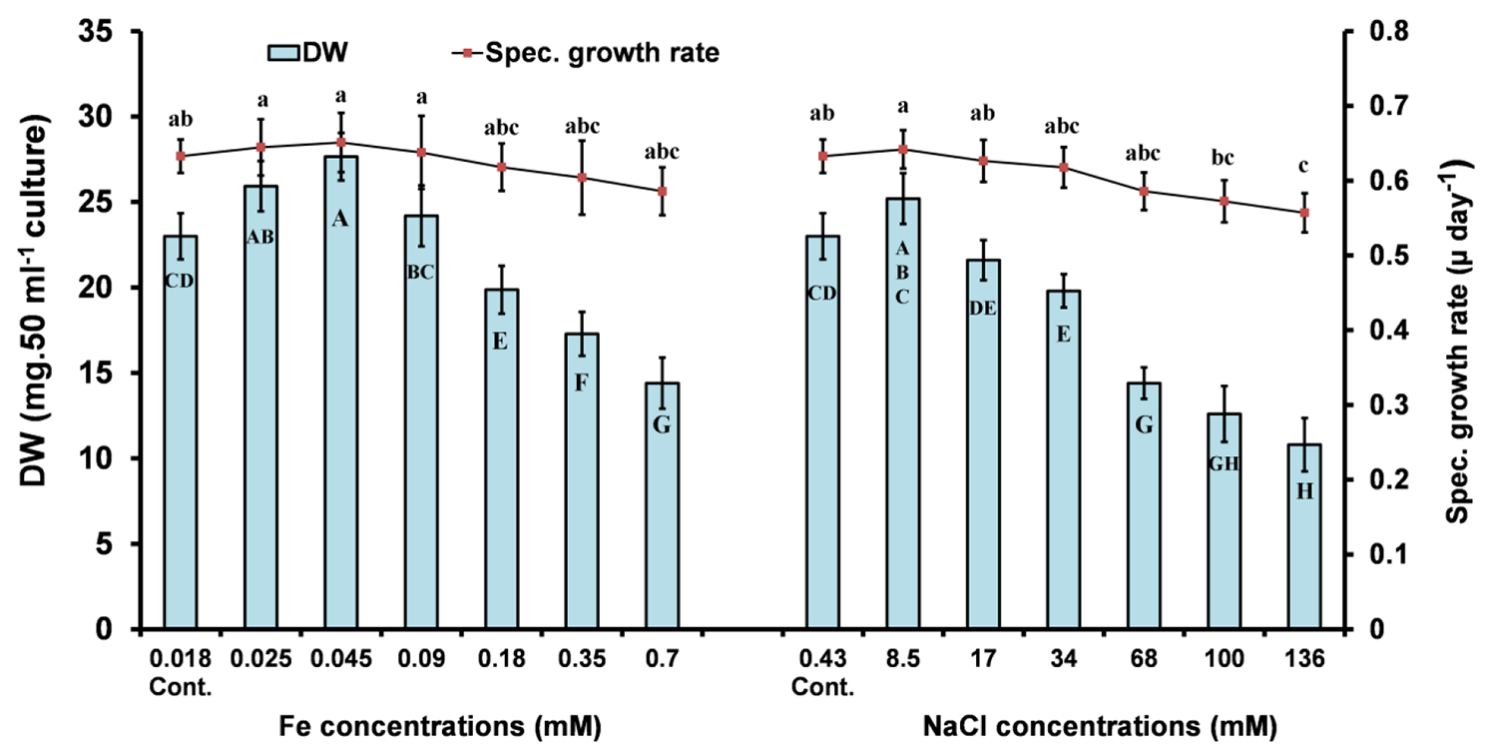
Biomass (dry weight; DW) and specific growth rate of *P. pringsheimii* under different concentrations of iron and salinity. The values represent the mean ± SD of three replicates. The different letters (for each parameter) represent significant differences at *P* < 0.05 (Duncan’s multiple range test)

Iron concentrations ranging from 0.025 to 0.09 mM were found to stimulate the biomass yield of *Ppr* by 5–20% above that of the control. However, concentrations above this range were found to be toxic. The specific growth rate of *Ppr* was slightly non-significantly (*P* < 0.05) higher (0.8-3%) at these concentrations (0.025 to 0.09 mM Fe). However, at higher Fe concentrations (up to 0.7 mM) the specific growth rate decreased to its lowest value (Fig. 1). On the other hand, the different concentrations of NaCl had an inhibitory effect on the biomass, and specific growth rate of the alga. However, at the first concentration (8.5 mM NaCl), an increase in dry weight and growth rate was observed (by 10 and 1.4%, respectively, compared to the control; Fig. 1).

The stimulatory effect of Fe (0.025–0.09 mM) and 8.5 mM NaCl on the biomass and growth rate of *Ppr* may reflect the need of these ions at such concentrations. This is likely due to the requirement of iron in electron transfer reactions, which are essential for photosynthesis, respiration, and nitrogen assimilation [11, 12]. However, an excess of iron ions beyond a critical concentration may have adverse effects on cellular homeostasis, cell membranes, and cellular components (such as proteins, lipids, DNA, etc.) if they are converted to ROS by Fenton or Haber-Weiss reactions or by stimulating their production (e.g., of hydroxyl radicals) [12]. In a related study, it was found that 10^− 2^ mM Fe stimulated the growth and specific growth rate of the green microalga *Tetradesmus obliquus* the most (0.36 ± 0.04 day^− 1^), followed by 10^− 4^ and 10^− 3^ mM, with the lowest cell densities reported at 10^− 1^ mM [12]. Wan et al. [11] also observed the stimulation of *Chlorella sorokiniana* growth rate at 10^− 1^, 10^− 2^, 10^− 3^, and 10^− 4^ mM Fe. This alga was unable to grow at Fe concentrations of 1 mM. In addition, cell densities, biomass, and growth rates were maximized by supplementing the culture medium with 10^− 2^ mM Fe. The biomass of *Scenedesmus dimorphus* gradually increased with different iron concentrations (0.16–0.8 mM), with the highest yield (1.75 ± 0.01 g.L^− 1^) obtained at the highest iron concentration studied (0.8 mM). The specific growth rate increased with iron concentrations up to 0.12 ± 0.01 day^− 1^ at 0.64 mM [23]. This suggests that the optimal iron level for growth varies for each species.

Regarding salinity stress, our findings are in agreement with those of Yun et al. [10], who observed a 1.3-fold increase in the biomass of *C. vulgaris* with 30 mM NaCl compared to the control. The increase in algal biomass may be attributed to enhanced photosynthetic activity, the accumulation of secondary metabolites, and the production of osmoregulators to withstand salinity stress [22]. However, at higher concentrations (45–200 mM NaCl), a gradual decrease in biomass was noticed. Similarly, Pandit et al. [22] found that adding up to 300 mM NaCl to media with *C. vulgaris* and *Acutodesmus obliquus* resulted in growth benefits, with the maximum benefit observed at 60 mM.

### Pigment and biochemical analyses

**Fig. 2:**
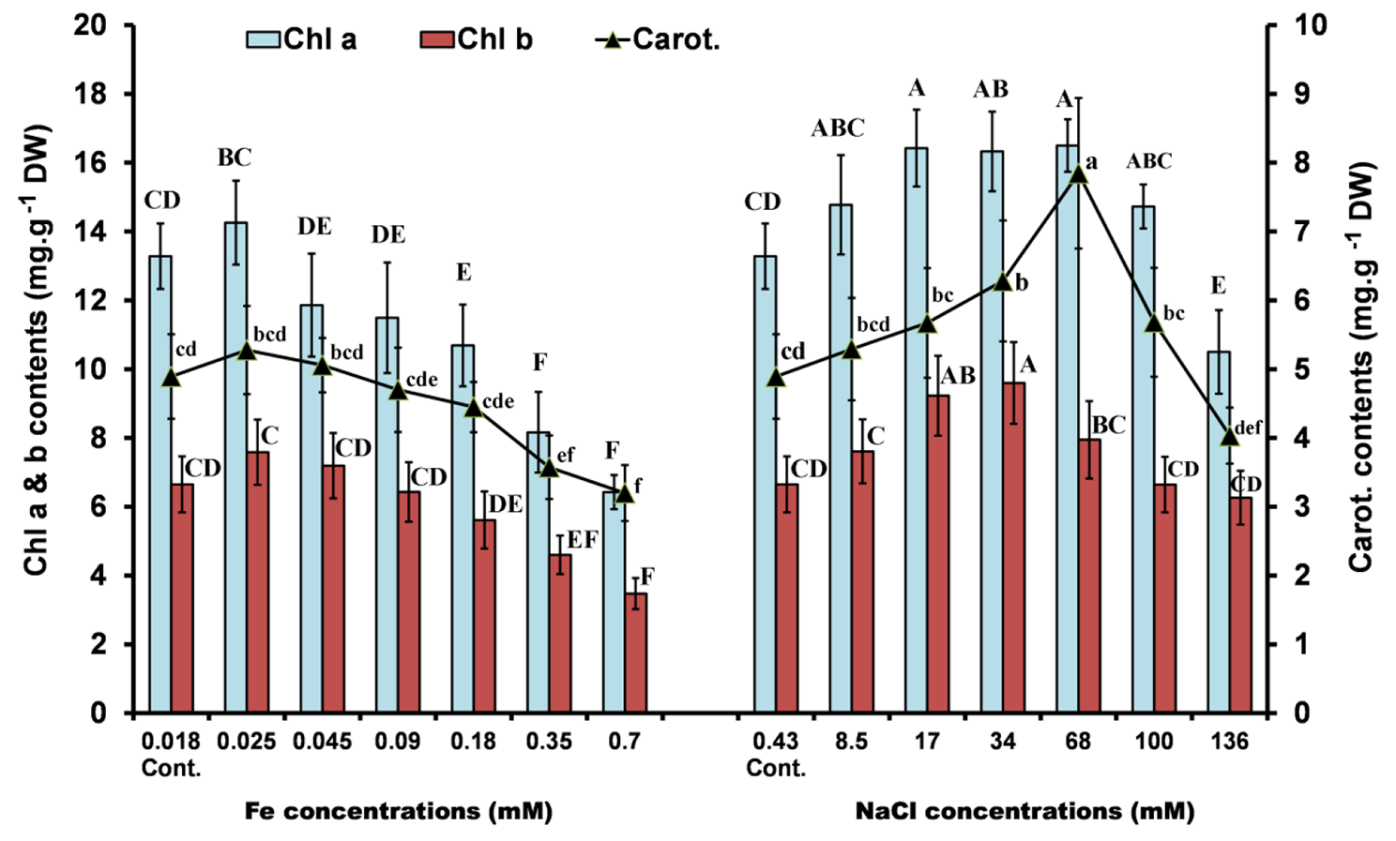
Chlorophyll a (Chl *a*), chlorophyll b (Chl *b*) and carotenoids contents of *P. pringsheimii* under different concentrations of iron and salinity. The values represent the mean ± SD of three replicates. The different letters (for each parameter) represent significant differences at *P* < 0.05 (Duncan’s multiple range test)

The pigment content of *Ppr* varied depending on the applied concentration of iron and NaCl. At a concentration of 0.025 mM Fe, the levels of Chl *a*, Chl *b*, and carotenoids were increased. Whereas, the higher concentrations had an inhibitory effect on the pigment fractions (Chl *a* & *b*, and carotenoids). On the other hand, all tested concentrations of NaCl increased the pigment levels, except for the highest concentration (136 mM NaCl). The maximum stimulation of Chl *a* (≈ 24% above the control) was detected in the range of 17 to 68 mM NaCl (Fig. 2).

Other studies have also confirmed this pattern. In *C*. *vulgaris* pigment production was found to increase at low levels of NaCl but was inhibited at higher concentrations [10, 22]. Pancha et al. [16] attributed the reduction in pigments to the degradation effect of NaCl as a direct response to the stressor. Moreover, the increased ROS accumulation may reduce the efficiency of algal photosynthesis due to its direct effect on lipid peroxidation of thylakoids and degradation of the PS II complex.

Considerable attention has been given to carotenoids, as they are known to be one of the most important non-enzymatic antioxidants used by algae to cope with oxidative stress. In addition to their function as accessory pigments in photosynthesis, they quench and reduce ROS accumulated by stressors [21]. In this study, the maximum stimulation of carotenoids was recorded at 0.025 mM Fe (≈ 8%) and 68 mM NaCl (60%) above control (Fig. 2). This increase may reflect the protective role of carotenoids which mitigates the effects of stress [44]. In this context, the carotenoids content of the green algae *Cladophora glomerata*, *Rhizoclonium crassipellitum*, *Pithophora cleveana*, and *Chaetomorpha aerea* were increased under salinity and N-stress until the 7^th^ day of treatment, after which the contents were decreased [1].

**Fig. 3:**
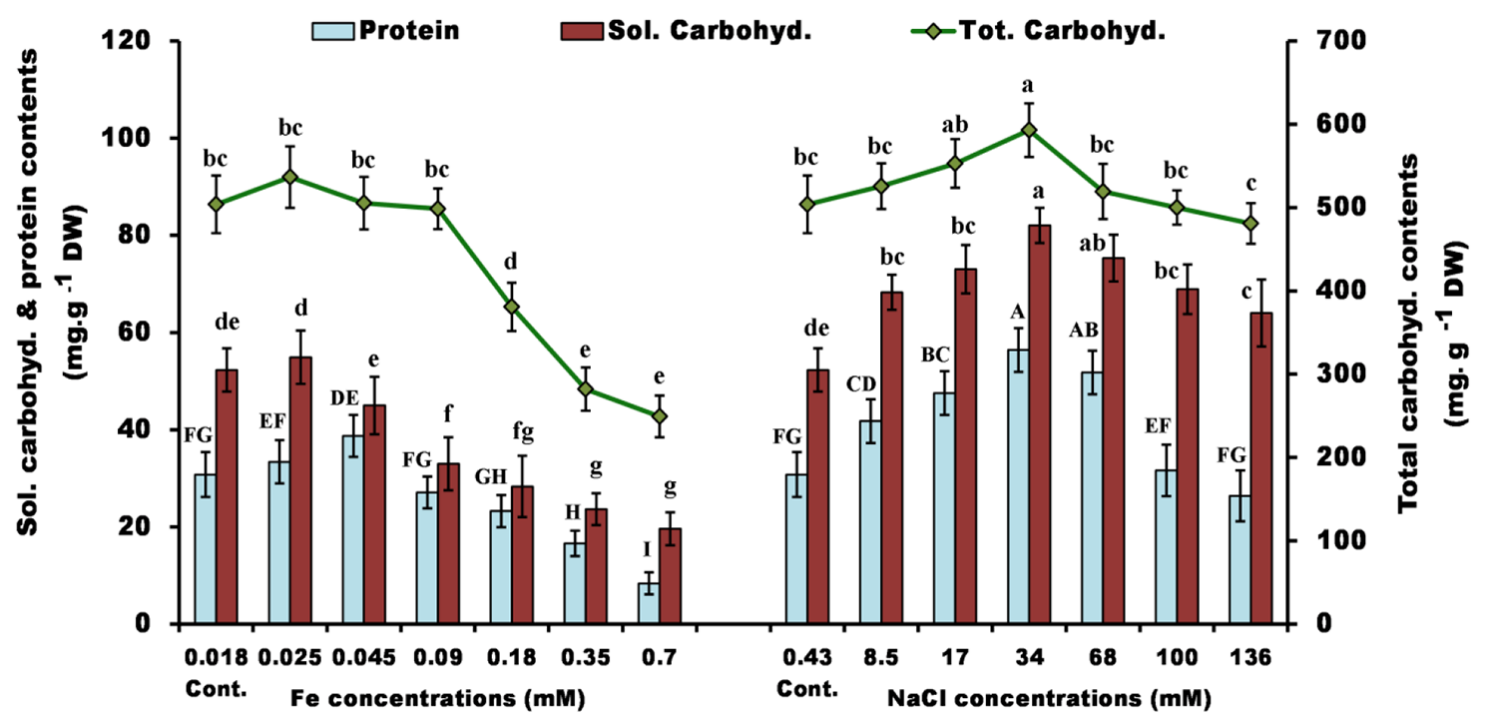
Total soluble protein, soluble and total carbohydrate contents of *P. pringsheimii* under different concentrations of iron and salinity. The values represent the mean ± SD of three replicates. The different letters (for each parameter) represent significant differences at *P* < 0.05 (Duncan’s multiple range test)

The protein contents of *Ppr* were also varied under the investigated stressors, with NaCl stress having a greater stimulatory effect than Fe. The range of 0.025 to 0.045 mM Fe had a stimulatory effect on protein levels (8.5–26% above the control), whereas all NaCl concentrations studied (except the highest dose; 136 mM) stimulated the protein content by 3–83%. The highest value was recorded at 34 mM NaCl (56.4 mg.g^− 1^ DW) followed by 68 mM NaCl (52 mg.g^− 1^ DW) compared to the control (31 mg.g^− 1^ DW) (Fig. 3).

The soluble and total carbohydrate fractions showed a similar trend as described above. In general, the iron concentrations had a strong inhibitory effect on the carbohydrate fractions compared with NaCl stress, however 0.025 mM Fe showed a non-significant (*P* < 0.05) stimulatory effect on the soluble and total carbohydrate contents (5 and 6.5%, respectively). For NaCl, the maximum stimulation was recorded at 34 mM (57 and 18% for soluble and total carbohydrates, respectively) (Fig. 3). The negative effect of iron treatment, compared to NaCl, may be attributed to the severe effects of the heavy metal on the metabolic pathways of algae. These include the impairment of the functional groups of important molecules such as enzymes, polynucleotides, active transport systems (for essential ions and nutrients), the enhancement of ROS production, which affects the activity of enzymes and the integrity of cellular membrane, and as a result, all cellular processes and homeostasis were affected [12, 45].

The content of protein and carbohydrates usually reflects the degree of stress imposed on the microorganism [22]. In response to stress, previous studies reported the increased accumulation of some specific biomolecules such as metal-binding proteins like phytochelatins, or other detoxifying proteins such as antioxidant enzymes, amino acids (which act as free radical scavengers), monosaccharides, disaccharides, and polysaccharides that protect cell membranes and other cellular components from the harmful effects of ions [7, 19, 21, 46].

The stimulation of carbohydrate content of *Ppr* was previously reported under the influence of Pb (up to 200 µM), whereas the metal Cd had a negative effect, especially at high levels [7]. Moreover, Pandit et al. [22] observed that increasing NaCl concentration (up to 0.4 M) increased the carbohydrate content of *C. vulgaris* (to 47% DW) and *A. obliquus* (to 51% DW) but decreased the protein content. They attributed this to the roles of carbohydrates, such as soluble sugars, in maintaining the osmotic regulation of algal cells. Yun et al. [10] reported the increased carbohydrate and decreased protein contents of *C. vulgaris* under elevated salinity stress (0-600 mM NaCl). Furthermore, the increased carbohydrate and decreased protein contents were also recorded in the green alga *Scenedesmus* sp. under salinity stress (up to 400 mM NaCl) [16]. To account for the enhancement of carbohydrates, Chokshi et al. [47] reported that under stress conditions, carbohydrates are considered a crucial storage component that can be used by living cells for their survival. However, further investigation of carbohydrate fractions under stress is advisable.

### Antioxidant activity

**Fig. 4:**
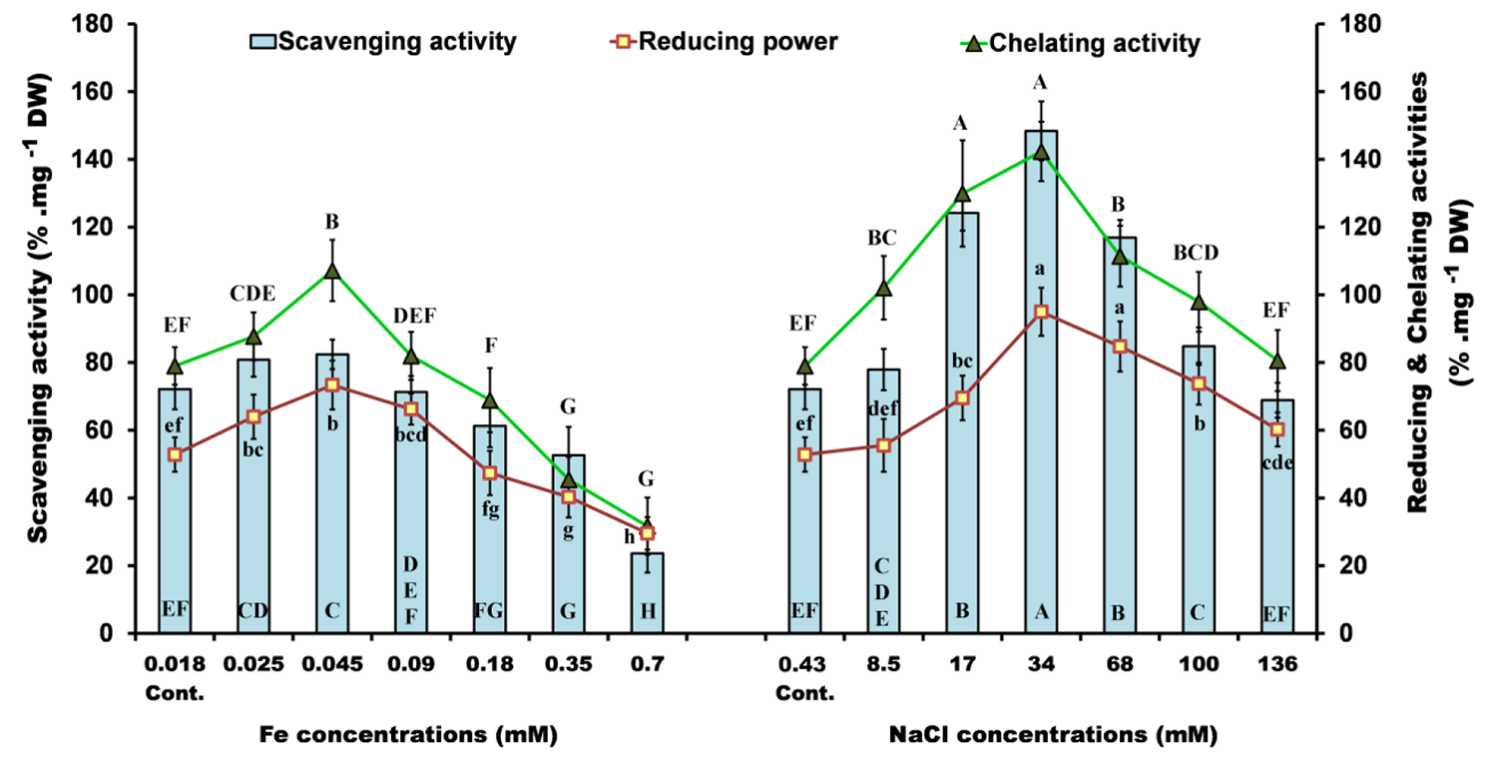
Antioxidant activity: Radical scavenging activity, reducing power and Fe(II) chelating activity of *P. pringsheimii* under different concentrations of iron and salinity. The values represent the mean ± SD of three replicates. The different letters (for each parameter) represent significant differences at *P* < 0.05 (Duncan’s multiple range test)

The antioxidant activities of the algal extracts are analogous to the synthetic antioxidant BHT. Overall, our results showed that antioxidant activity was increased by lower concentrations of iron (0.025–0.09 mM) and by all of the NaCl concentrations (8.5–136 mM) (Fig. 4).

Based on the investigated characteristics, the algal extracts, proved the ability to stabilize free radicals by donating hydrogen donation, blocking radical chain reactions, and mitigating the harmful effects on algal cells under stress. This may be attributed to the significant biochemical components of the investigated alga such as chlorophylls, carotenoids, phenolics, polysaccharides, amino acids, proteins, and polyunsaturated fatty acids which may help alleviate the harmful effects of these ions [4, 5, 7, 21, 48].

The high antioxidant activity of *Ppr* under salinity compared to iron stress may be related, as discussed above, to the enhancement of significant biochemical components, and/or the induction of specific antioxidant components to alleviate the harmful effect of stressors. Moreover, the high value of the antioxidant activity of *Ppr* under salinity may be attributed to the good adaptation to NaCl ions rather than iron. This highlights the serious toxicity of iron, as a heavy metal, when applied in high concentrations.

A previous study found that *Dunaliella salina* exhibited increased free radical (DPPH) scavenging and reducing power activities under salinity, temperature, and nitrogen stresses [5]. Additionally, the effects of heavy metals (Cd and Pb) on *Ppr* have been shown to enhance the antioxidant capacity of the alga, with the exception of higher concentrations [7].

The high antioxidant values (Fig. 4) may be attributed to the content of algal carotenoids as there is a strong association between these activities and carotene production under such stress conditions [5]. In addition, the DPPH scavenging and reducing power activities of the green algae *Cladophora glomerata*, *Rhizoclonium crassipellitum*, *Pithophora cleveana*, and *Chaetomorpha aerea* were enhanced under salinity and N stresses. These antioxidant activities were shown to be proportional to the total phenolic content of the investigated algae [1]. The activities of DPPH scavenging and reducing power (Fig. 4) were mainly dependent on the contribution of carotenoids, while the phenolic contents were contributors to all algal antioxidants measured assays [4]. Furthermore, the polysaccharides and protein fractions of *Chlorella pyrenoidosa* proved their antioxidant activity through the DPPH radical scavenging, chelating, and reducing power activity tests [48, 49].

### Proteome changes under stress

The results of 1D SDS-PAGE analysis revealed a similar protein profile for the Fe and NaCl samples. Interestingly, two bands of ≈ 51 and 33 kDa were differentially expressed under salinity stress (Sup. Fig. S1). For more details, the protein samples were separated by 2D-PAGE. A total of 67 differentially abundant protein spots were recognized, of which the spots number 31–44 (except 43) and 45–67 were unique under iron and salinity stress, respectively (Sup. Fig. S2-S7).

**Fig. 5:**
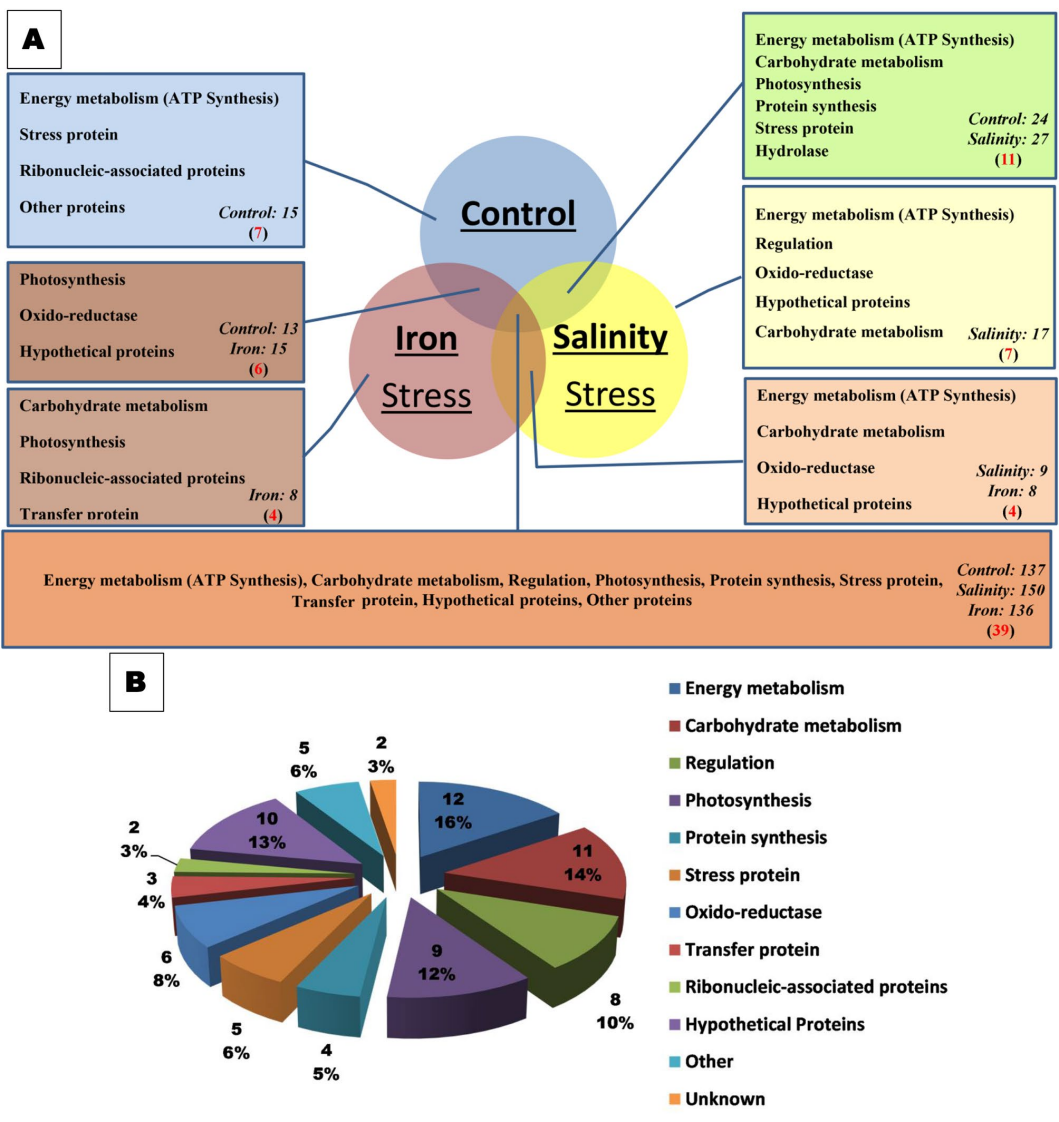
**A**. Venn diagram representation of the number of differently abundant peptides identified by the gel-based approach, the name of protein class, and their number (in parentheses) of *P. pringsheimii* under the investigated stressors. **B.** Total functional classification of the 77 proteins detected by the comparative 1D proteome analysis of the selected samples under stressors. The values represent the number of the identified protein and their percentage for each class

The LC-MS analysis of the 1D gel-proteins revealed 559 peptides, corresponding to 77 protein-groups (Sup. Table S1). Out of these peptides, 15 were unique to the control, 8 were unique to the iron, and 17 peptides were unique to the salinity treatment (Fig. 5A). Moreover, 39 common proteins were differentially regulated by both treatments, with 150 peptides showing increased abundance after salinity stress, compared to 136 of iron, and 137 for the control (Fig. 5A).

**Fig. 6:**
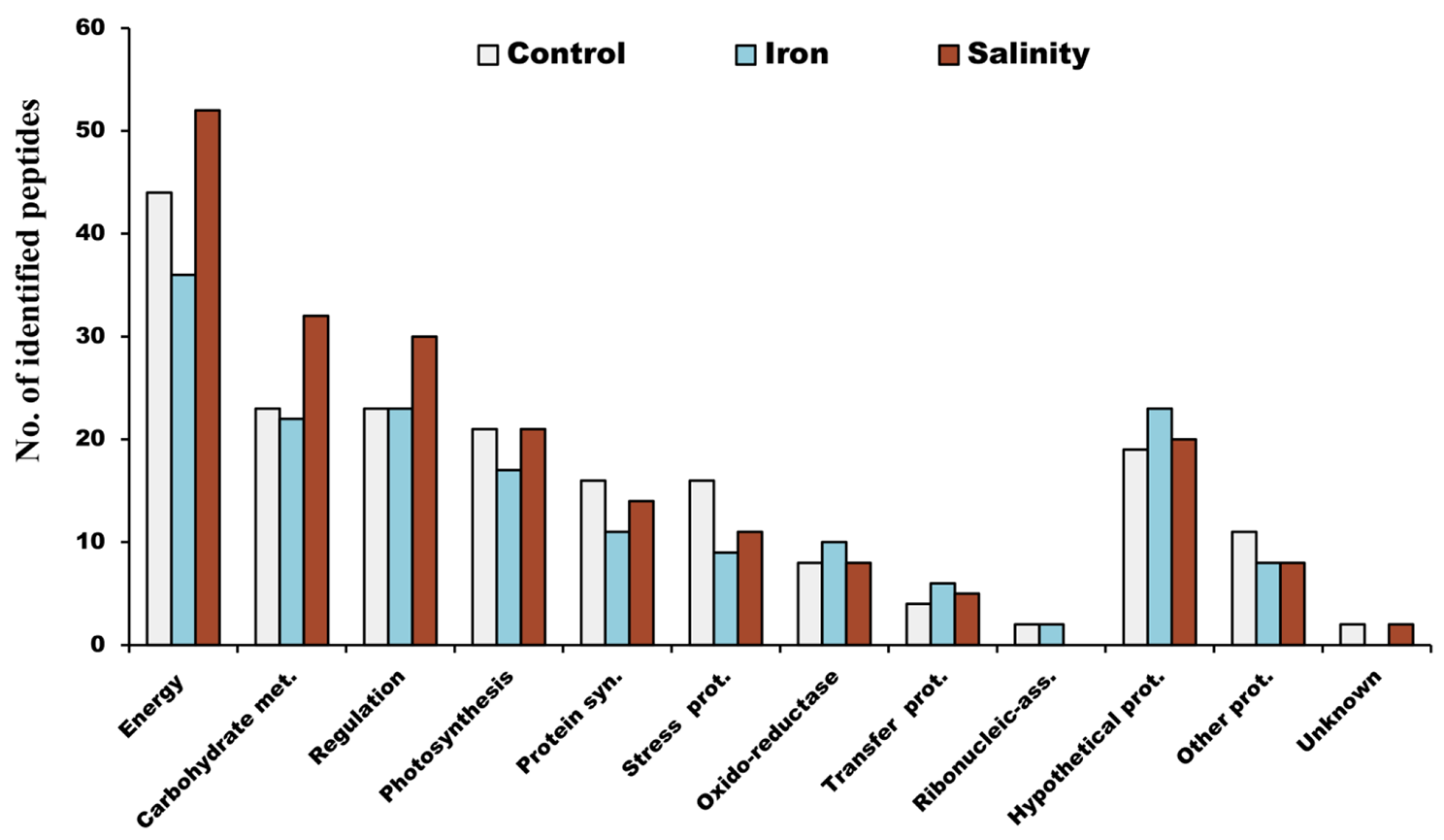
Number of the differently abundant peptides of *P. pringsheimii* grouped by protein classes under the stressors studied

The 77 identified proteins were classified into 12 functional categories: energy metabolism (ATP synthesis; 12 proteins), carbohydrate metabolism (11 proteins), regulation (8 proteins), photosynthesis (9 proteins), protein synthesis (4 proteins), stress proteins (5 proteins), oxido-reductase proteins (6 proteins), transfer proteins (3 proteins), ribonucleic-associated proteins (2 proteins), hypothetical proteins (10 proteins), other (5 proteins) and two unknown proteins (Fig. 5B). The differentially abundant peptides were grouped under these 12 categories (Fig. 6).

**Fig. 7:**
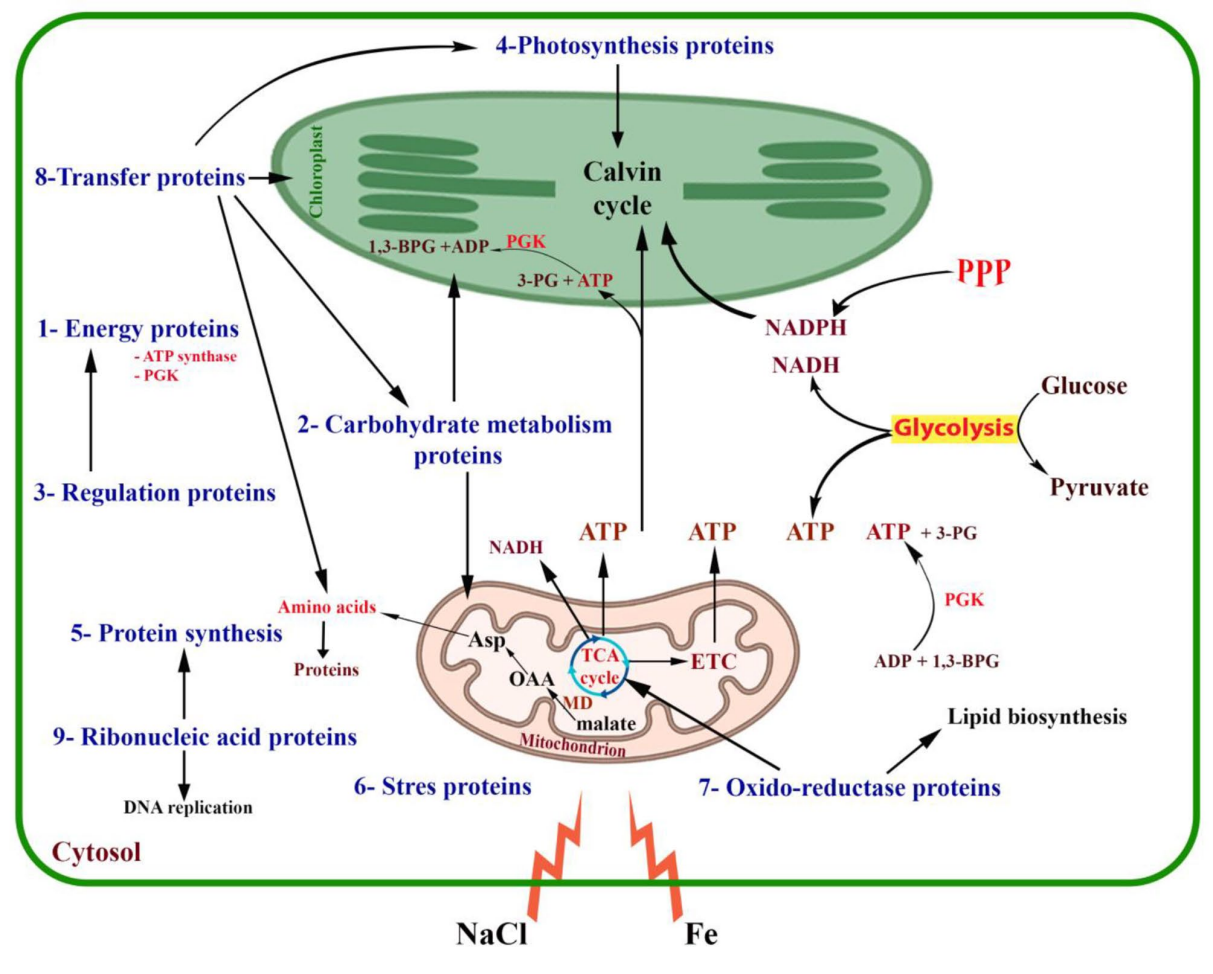
Schematic representation of the role and interplay of the differentially abundant proteins of *P. pringsheimii* under the stressors studied. **PPP**: pentose phosphate pathway, **PGK**: phosphoglycerate kinase, **1,3-BPG**: 1,3-bisphosphoglycerate, **3-PG**: 3-phosphoglyceric acid, **Asp**: aspartate; **OAA**: oxaloacetate, **MD**: malate dehydrogenase, **TCA**: the tricarboxylic acid cycle, **ETC**: electron transport chain

As depicted in Fig. 7, iron and salinity stressors affect the algal cells by stimulating certain cellular proteins. The most notable response was an increase in ATP production, primarily due to an increase in ATP synthase abundance, which provides energy for various cellular metabolic pathways. Proteins involved in growth (cell division), nucleic acid maintenance, carbohydrate metabolism, cellular regulation, protein synthesis, photosynthesis, etc. also contribute to stress tolerance. Furthermore, oxido-reductase proteins are crucial for the TCA cycle and lipid biosynthesis. Further discussion is provided below:

### Energy and carbohydrate metabolism

The majority of identified peptides were found to belong to the class of energy metabolism (Fig. 6, Sup. Table S1). It is not surprising that the algal cells attempted to increase their energy levels in order to cope with the stress (Fig. 7). This could be attributed to the high energy expenditure for metabolic activities under stress, as well as for the synthesis of biologically important molecules (such as phytochelatins and glycine) which are used as protectants and osmoregulants to maintain cellular homeostasis [3, 22]. Similar to our findings, the expression of different ATP synthase subunits (α and β) in *D. salina* and *C. reinhardtii* has been reported under salinity stress [26, 27, 29].

We have also observed several significant peptides related to fructose-1,6-bisphosphatase (FBPase), sedoheptulose-1,7-biphosphatase (SBPase), ribulose-1,5-bisphosphate carboxylase/oxygenase (Rubisco), glyceraldehyde-3-phosphate dehydrogenase (GAPDH), and enolase (phosphopyruvate hydratase) which showed increased abundance particularly under salinity stress (Fig. 6, Sup. Table S1). This finding is in line with previous reports in *Anabaena doliolum*, *C*. *sorokiniana*, *Phaeodactylum tricornutum*, and *Sargassum fusiforme* under iron, Cd, and salinity stress [3, 11, 15, 25, 50]. Moreover, proteins such as Rubisco, FBPase, GAPDH, SBPase, and other thiol-modulated enzymes of the chloroplast play important roles during oxidative conditions [18, 51, 52]. However, beyond a certain level of stress, and depending on the tolerance of the species tested, a reduction in the abundance of these proteins may occur [3, 27, 50, 53].

### Regulation

The data revealed that the number of regulatory peptides of *Ppr* remained constant under iron stress; however, the number increased under salinity stress (Fig. 6, Sup. Table S1). These peptides play a crucial role in mitigating stress and properly modulating cell functions. For instance, the membrane AAA-metalloprotease, which was almost universally identified among the stressors studied, serve numerous cellular functions by providing energy through ATPase activity. In addition, it aids in repairing or removing damaged and unnecessary proteins to maintain healthy and functional mitochondria, especially under stress conditions [54]. This was previously reported in salt-shocked cells of *C*. *reinhardtii* [24] and *D. salina* [28].

Moreover, the identified chaperonin peptides (Sup. Table S1) have been shown to facilitate the folding of cellular proteins into their active form and preserve their integrity, particularly in stressful conditions [55]. In this regard, several chaperonin subunits were differentially increased under salinity stress in *C. reinhardtii* [24], *D. salina* [28], and many other plant species [52], highlighting their crucial role in maintaining cellular homeostasis under stress.

An alpha-tubulin peptide was identified only under salinity stress, whereas the beta-subunit peptides were found to be more abundant under both stressors, compared to the control. The number of identified actin peptides remained constant under control and stress conditions (Sup. Table S1). Tubulin was found to increase in response to heavy metals and salinity stressors in *D. salina*, *Chlamydomonas* sp., and *C. reinhardtii*, where it binds and stabilizes the plasma membrane [26, 29, 53]. However, Jia et al. [27] observed a decrease in the α- and β-tubulin subunits in *D*. *salina* under high salinity stress.

Another regulatory protein, S-Adenosyl homocysteine (SAH) hydrolase, has been identified under salinity stress (in addition to the control sample; Sup. Table S1). It has been found to be differentially expressed under cold stress in *S. platensis* [20]. We believe that this protein is crucial for *Ppr* tolerance as it plays a vital role in various cellular processes, including the regulation of the methylation reaction, and amino acid metabolism. This includes the production of homocysteine, which is considered a detoxification mechanism for heavy metal stress [56].

### Photosynthesis

The number of peptides associated with the photosynthesis process in *Ppr* under salinity was the same as that of the control; however, the number was low under iron stress (Fig. 6, Sup. Table S1). These findings may be attributed to the fact that stress conditions typically have a negative impact on the photosynthetic process, leading to damage to the algal cell and eventual death. This could be related to the direct effect of stress on the biosynthesis of proteins related to photosynthesis, resulting in their downregulation. Carbonylation, phosphorylation/dephosphorylation, and redox changes of thiol groups are possible pathways for these modifications [3, 52]. A similar pattern of results was obtained for the photosynthetic proteins in *C*. *sorokiniana*, *C*. *reinhardtii*, and *Chlamydomonas* sp. under heavy metals and salinity stress [3, 19, 25, 53].

### Protein synthesis

The low abundance of peptides related to protein synthesis was expected due to the reduced protein contents at such doses of stressors. Notably, the number of peptides was lower under iron stress compared to salinity stress (Fig. 6), which was consistent with protein content (Fig. 3). In general, the differentially expressed protein synthesis -peptides were found to play an important role under stress [29]. Among these peptides, the eukaryotic initiation, and elongation factors (Sup. Table S1) were identified in the salinity-tolerant strain *C*. *reinhardtii* [24, 28]. Moreover, the eukaryotic initiation, elongation, and translation factors (Sup. Table S1) were also identified in the halotolerant strain *D. salina* [29].

The peptides of translation elongation factor Tu of *Ppr* remained constant under the stressors (Sup. Table S1),reflecting their importance in facilitating peptide bond formation in protein synthesis under these conditions [57].

### Stress proteins

The proteomic analysis of *Ppr* revealed the presence of various stress proteins that could be responsible for adaptation to the stressors studied (Fig. 6, Sup. Table S1). Some of these peptides, such as heat shock protein (HSP) 70, HSP 90, and luminal binding protein Bip1, play an important role in anti-stress mechanisms. However, the thioredoxin/transketolase fusion protein and peroxiredoxin were only identified in the control sample (Sup. Table S1). The main function of stress proteins is to protect cellular components, maintain cellular metabolism, and eliminate damaged components under stress conditions [6, 58].

The detected HSP 70 (~ 70 kDa) and 90 (~ 90 kDa) (Sup. Table S1) are chaperone proteins that assist in protein folding in cells under normal and stress conditions [6, 58]. The genes of HSP 70 are mainly activated by environmental stress and toxic substances, making them promising biomarkers for ecotoxicogenomics assessment in various organisms [6, 53, 59].

The luminal binding protein BiP1 was consistently present in both control and treated samples (Sup. Table S1). It is another chaperone protein that aids in the import and translocation of cellular proteins and is believed to facilitate the assembly of multimeric protein complexes that confer stress tolerance upon expression [6, 28].

### Oxido-reductase proteins

The abundance of oxido-reductase peptides was increased under iron stressor, but remained constant under salinity stressor compared to the control (Fig. 6). Specifically, different peptides of the malate dehydrogenase protein (MDH) were identified among the stressors studied (Sup. Table S1). As a conserved TCA cycle enzyme, MDH plays an important role in various metabolic pathways (Fig. 7) and was therefore differentially expressed by different photosynthetic plants under cadmium stress [9, 50].

The enoyl-ACP reductase protein, involved in lipid biosynthesis, was identified under the iron stressor in addition to the control sample (Sup. Table S1). Previously, the induction of enoyl-ACP reductase was also observed with iron treatment of the diatom *Phaeodactylum tricornutum* [25]. It was also identified in the proteomics of *C. reinhardtii* control (progenitor) cells, while it was not detected under salinity (300 mM NaCl) stress [29]. However, the lipid contents of *Ppr* should be investigated to determine its role, especially under stress.

### Transfer proteins

The transfer proteins were found to be more abundant under stressors compared to the control (Fig. 6). These proteins, as discussed below, play a crucial role in facilitating the transfer of various groups and molecules for the benefit of the cell (Fig. 7).

One such protein, phosphoribulokinase (PRK), was consistently present in the treatments (Sup. Table S1). PRK is an essential ATP-dependent transferase enzyme for photosynthesis, catalyzing the transfer of a phosphorus group to convert ribulose-5-phosphate (RuP) into RuBP, which is important for the Calvin cycle and carbon fixation in autotrophic organisms [52]. Previous proteomic analyses have identified PRK in various organisms under different stress conditions, such as the cyanobacterium *S. platensis* under iron and salinity stresses [18], the green algae *C*. *reinhardtii* and *D. salina* under salinity stress [24, 28, 29], *Chlamydomonas* sp. under heavy metal stress [53], and the diatom *Phaeodactylum tricornutum* under iron stress [25]. Moreover, differential expression of the PRK protein has been observed in many crop plants in response to varying levels of salinity stress [52]. However, downregulation of PRK under salinity stress has been reported in the cyanobacterium *Anabaena doliolum*, which directly affects the availability of NADPH for further cellular processes [15]. This suggests that the expression level of PRK and other cellular proteins may depend on the adaptability of the species to different types of stress, concentration of the stressors, and mode of action of the applied stressors, as well as the upregulation of other cellular pathways such as that of the RuBisCO induction [15].

Aspartate aminotransferase (AST) is another transfer protein, that is consistently detected constantly under the investigated stressors (Sup. Table S1). AST plays an important role in catalyzing the reversible transamination of glutamate and oxaloacetate to produce aspartate and 2-oxoglutarate which is crucial for C and N metabolism. Most importantly, the transferred amino groups between amino acids play a role in saving reductants and constituent proteins for proper growth under both normal and stress conditions [60, 61]. Accumulation of AST (2.3-fold increase) has been reported in *C*. *sorokiniana* under Cd stress indicating its importance for glutamate and cysteine metabolism as a detoxification mechanism for heavy metal stressors [3]. It was also upregulated in *D. salina* under high salinity stress [27], and in plants subjected to heavy metal stress [9].

Dihydrolipoamide acetyltransferase (E2) was only identified under iron stress (Sup. Table S1), which catalyzes the transfer of the acetyl group to CoA to produce acetyl-CoA. Correspondingly, acetyl-CoA is a biologically important molecule that takes part in a variety of metabolic reactions involved in protein, carbohydrate, and lipid metabolism [17].

### Ribonucleic-associated proteins

Four peptides related to nucleic acid processing (or structure) were identified in *Ppr* under iron stressor (viz. RNA helicase), and in the control sample (ribosomal protein) (Sup. Table S1). Plastid ribosomal protein L1 is associated with the structural constituent of the ribosome for protein synthesis, while RNA helicase unwinds the double helix of DNA to initiate its replication (Fig. 7).

### Hypothetical, unknown, and other proteins

A number of peptides related to hypothetical and other proteins were identified in *Ppr* under stressors. In addition, other unannotated proteins with unknown functions were also detected (Sup. Table S1).

## Conclusion

The current study has discussed the response of the green alga *P. pringsheimii* to two environmental stressors; iron and salinity. Multiple approaches including proteomic, biochemical, and physiological methods were employed to comprehend the survival of the microalga under the studied stressors. The biomass, growth, biochemical contents, and antioxidant activity of the alga were reported under these stressors. The lower concentrations of the stressors had a stimulatory effect on the growth and biochemical content, while higher concentrations had an inhibitory effect on these parameters. Iron concentrations, except for the lower ones, had a strong inhibitory effect on the protein and carbohydrate fractions compared to the salinity. In addition, the high antioxidant activity was more prominent under salinity compared to iron stressor. The proteomic analysis of *P. pringsheimii* revealed a total of 559 peptides corresponding to 77 proteins, which were functionally classified into 12 different categories. The majority of the proteins were involved in ATP generation, followed by carbohydrate metabolism, and photosynthesis which emphasized the important role of these proteins under stress. Proteins related to growth, Calvin cycle, nucleic acid maintenance, stress tolerance, etc. proved their essential existence under both stressors. The salinity (136 mM NaCl) was found to have more of the identified peptides than control or iron treatment (0.35 mM Fe). The data obtained from this study may be useful for future genetic and proteomic analysis of microalgae under other environmental and chemical stresses. Moreover, it could confer a database for understanding the tolerance and better exploitation of microalgae under stress. Further investigation into the expression of genes related to the differentially expressed proteins is recommended.

### Electronic supplementary material

Below is the link to the electronic supplementary material.

Supplementary Material 1

Supplementary Material 2

## Data Availability

Further data that support the findings of this study are available on request from the corresponding author.
