# Supplementary material for: Biochemical and proteomic response of the freshwater green alga Pseudochlorella pringsheimii to iron and salinity stressors
Source: BMC Plant Biol. 2024 Jan 10;24:42. doi: 10.1186/s12870-023-04688-9 (PMC10777535; doi:10.1186/s12870-023-04688-9)
Supplement: Supplementary file 1 — Supplementary Material 1 [file 12870_2023_4688_MOESM1_ESM.pdf]

Supplementary materials

- Journal name: **BMC Plant Biology**

- Manuscript Title: **Biochemical and proteomic response of the freshwater green alga *Pseudochlorella pringsheimii* to iron and salinity stressors**

Authors names: **Mostafa M. S. Ismaiel[1*], Michele D. Piercey-Normore[2], Christof Rampitsch[3]**

[1] Department of Botany and Microbiology, Faculty of Science, Zagazig University, Zagazig, 44519, Egypt.

[2] Faculty of Science, Algoma University, Sault Ste Marie, Ontario, P6A 2G4, Canada.

[3] Agriculture and Agri-Food Canada, Morden Research and Development Centre, Morden MB, Canada R6M 1Y5.

* Corresponding author; e-mail mostafamsami@yahoo.com; permanent address: Botany Department, Faculty of Science, Zagazig University, Zagazig, 44519, Egypt.

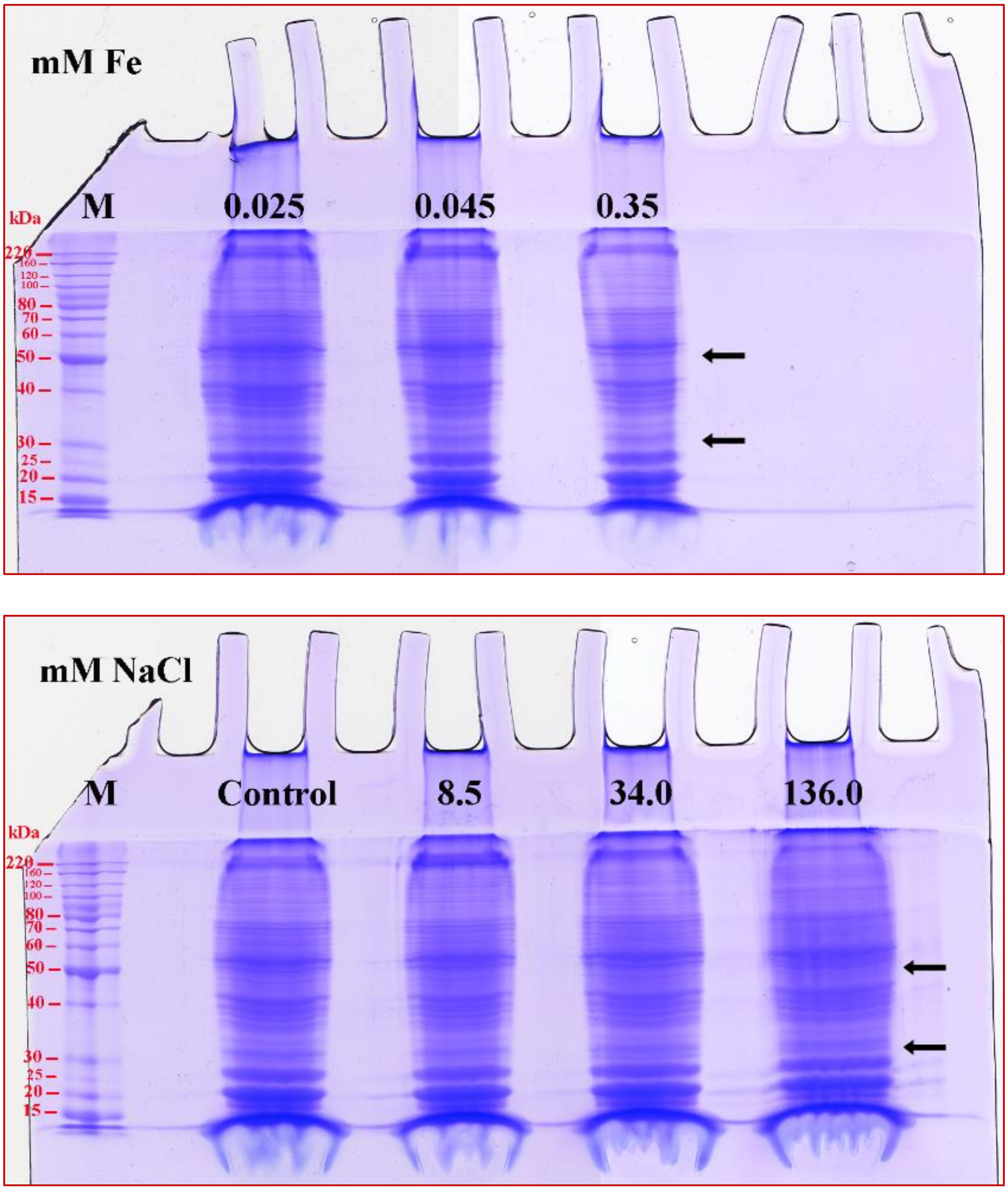

Fig. S1. 1-D proteomics analysis by of *Pseudochlorella pringsheimii*. Proteins (20 µg /lane) were analyzed by SDS-PAGE (12%) and stained with Coomassie® Brilliant Blue R250. M: BenchMark™ Protein Ladder (Invitrogen, CA, USA). Control (0.018 mM Fe & 0.43 mM NaCl). Other treatments are indicated above each lane: 0.025 mM Fe; 0.045 mM Fe; 0.35 mM Fe; 8.5 mM NaCl; 34.0 mM NaCl; and 136.0 mM NaCl.

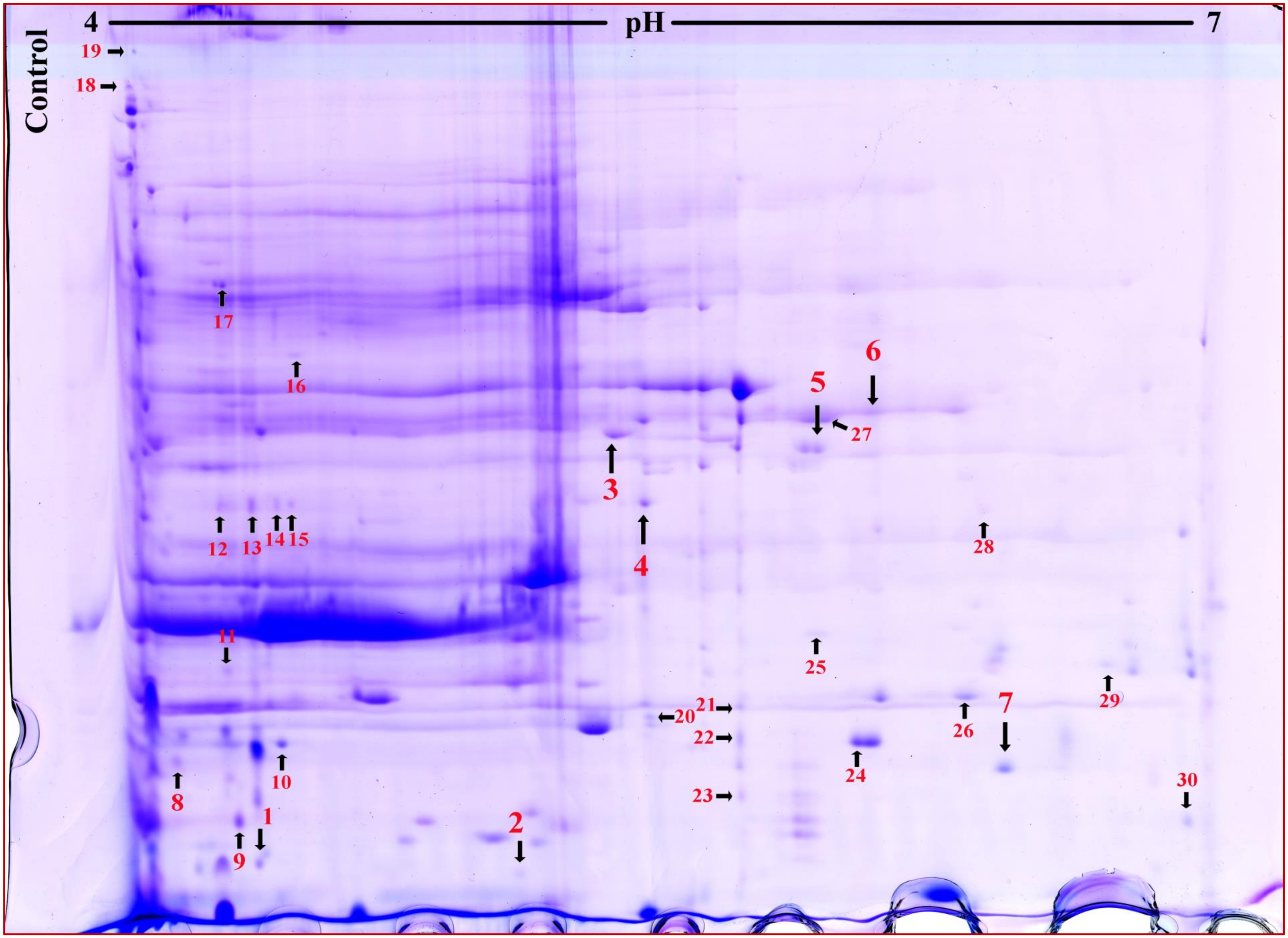

Fig. S2. A representative 2D-gel electrophoresis gel of *P. pringsheimii* total protein under **control condition (0.018 mM Fe & 0.43 mM NaCl)**. 550 µg of sample were loaded onto a 4-7 PI linear IPG strip and subject to isoelectric focusing (IEF) in the first dimension. The IPG strips were then loaded on a 12% SDS-PAGE gel for separation of proteins by size in the second dimension. Proteins were stained with Coomassie Blue.

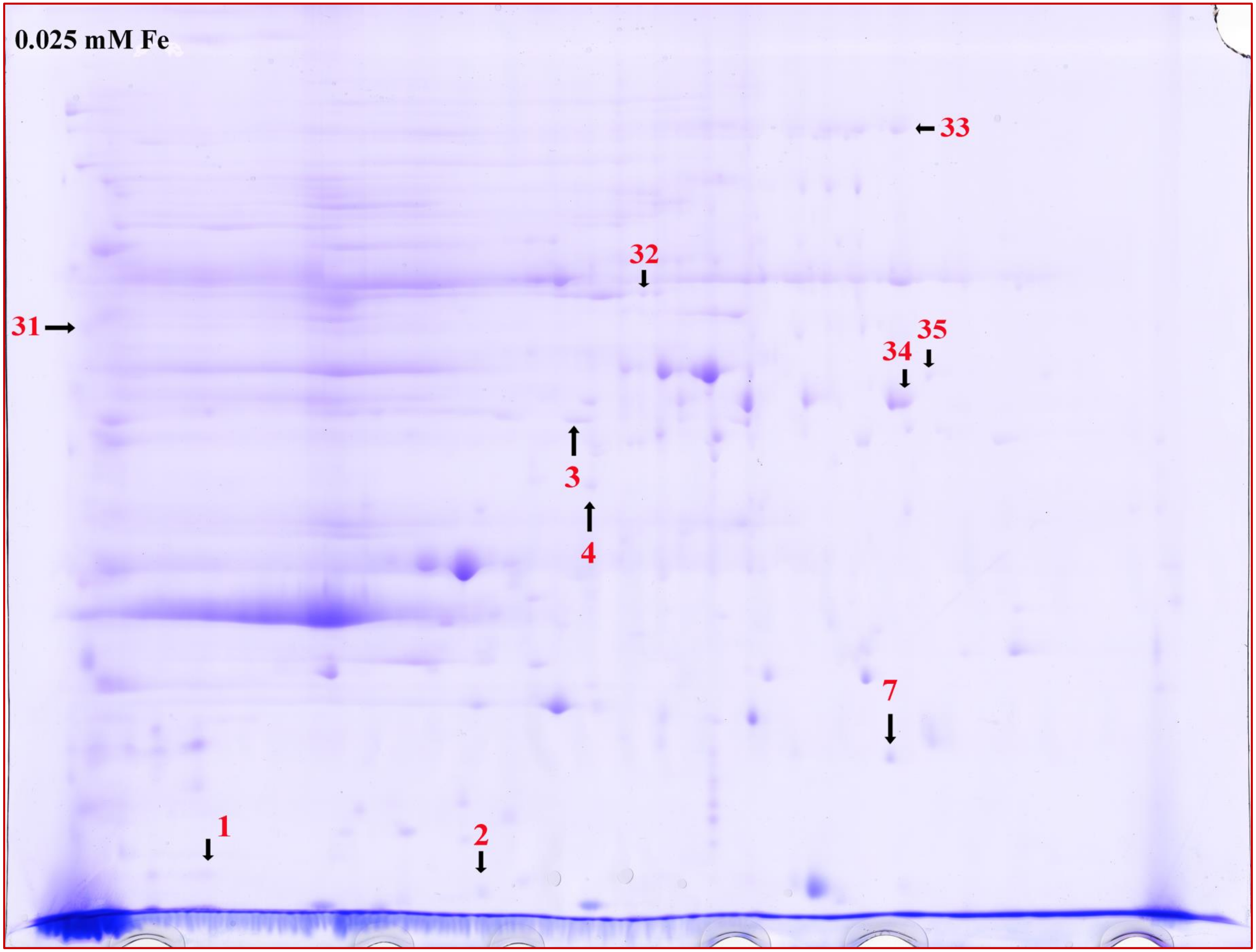

Fig. S3. A representative 2D-gel electrophoresis gel of *P. pringsheimii* total protein under **0.025 mM iron stress**. 550 µg of sample were loaded onto a 4-7 PI linear IPG strip and subject to isoelectric focusing (IEF) in the first dimension. The IPG strips were then loaded on a 12% SDS-PAGE gel for separation of proteins by size in the second dimension. Proteins were stained with Coomassie Blue.

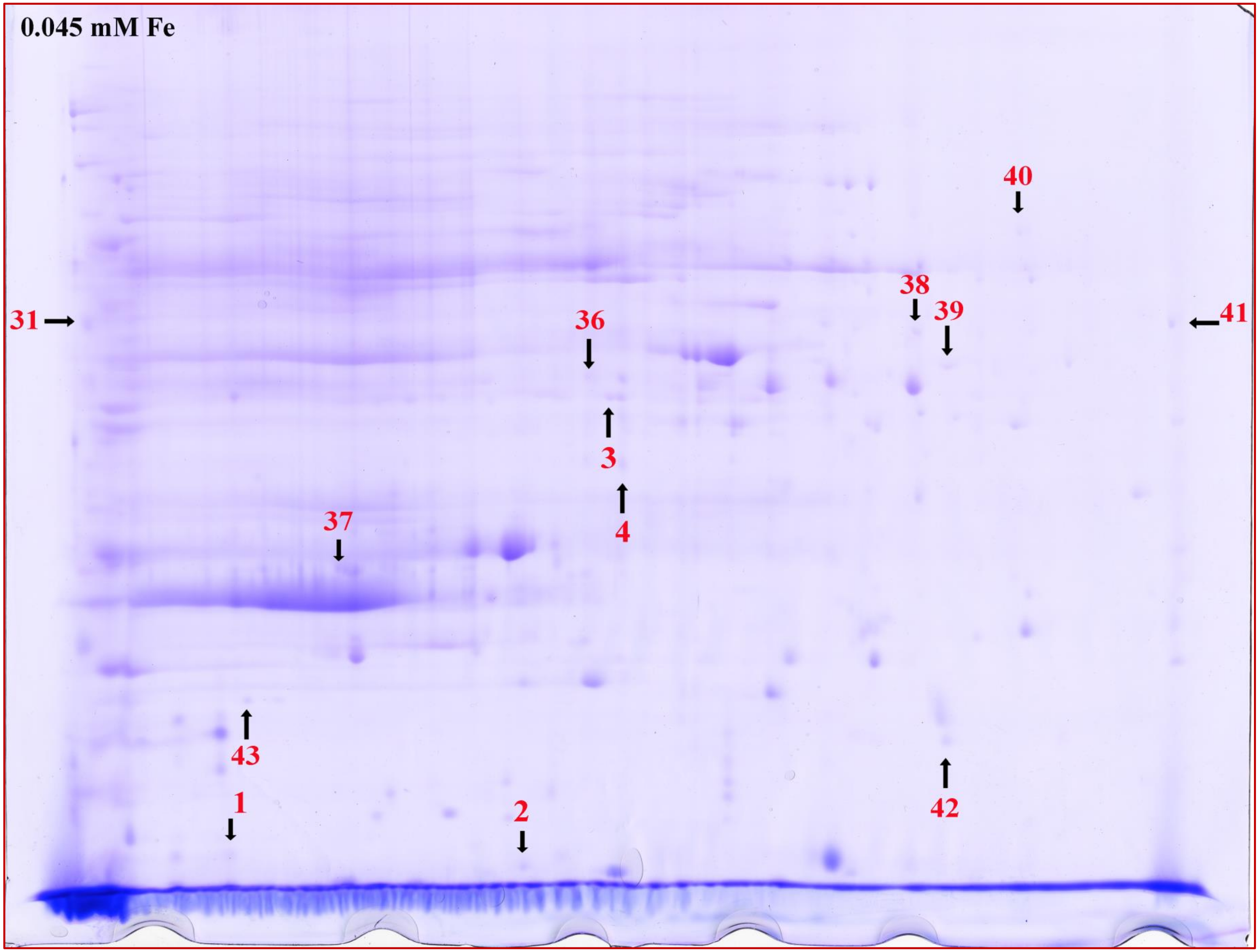

Fig. S4. A representative 2D-gel electrophoresis gel of *P. pringsheimii* total protein under **0.045 mM iron stress**. 550 µg of sample were loaded onto a 4-7 PI linear IPG strip and subject to isoelectric focusing (IEF) in the first dimension. The IPG strips were then loaded on a 12% SDS-PAGE gel for separation of proteins by size in the second dimension. Proteins were stained with Coomassie Blue.

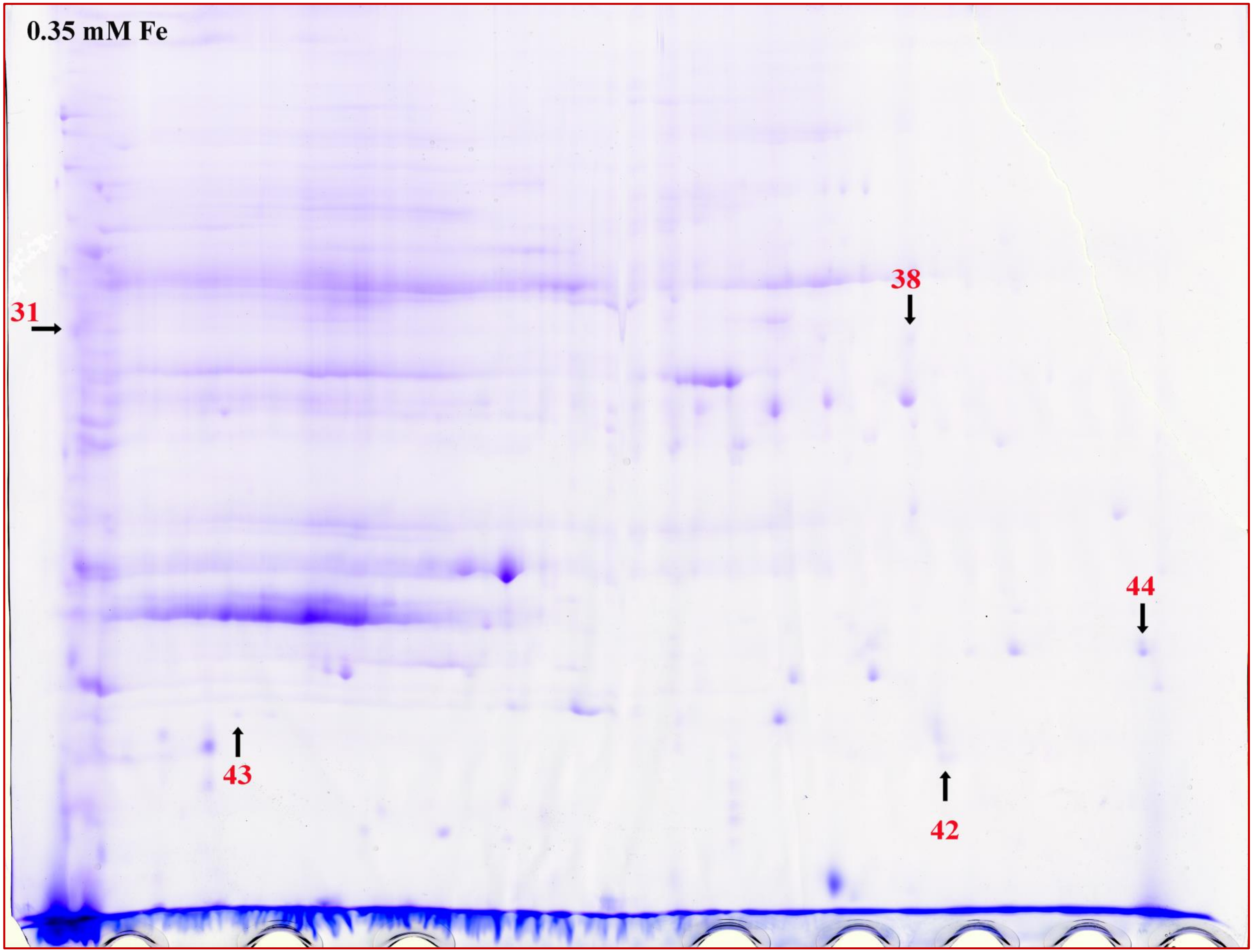

Fig. S5. A representative 2D-gel electrophoresis gel of *P. pringsheimii* total protein under **0.35 mM iron stress**. 550 µg of sample were loaded onto a 4-7 PI linear IPG strip and subject to isoelectric focusing (IEF) in the first dimension. The IPG strips were then loaded on a 12% SDS-PAGE gel for separation of proteins by size in the second dimension. Proteins were stained with Coomassie Blue.

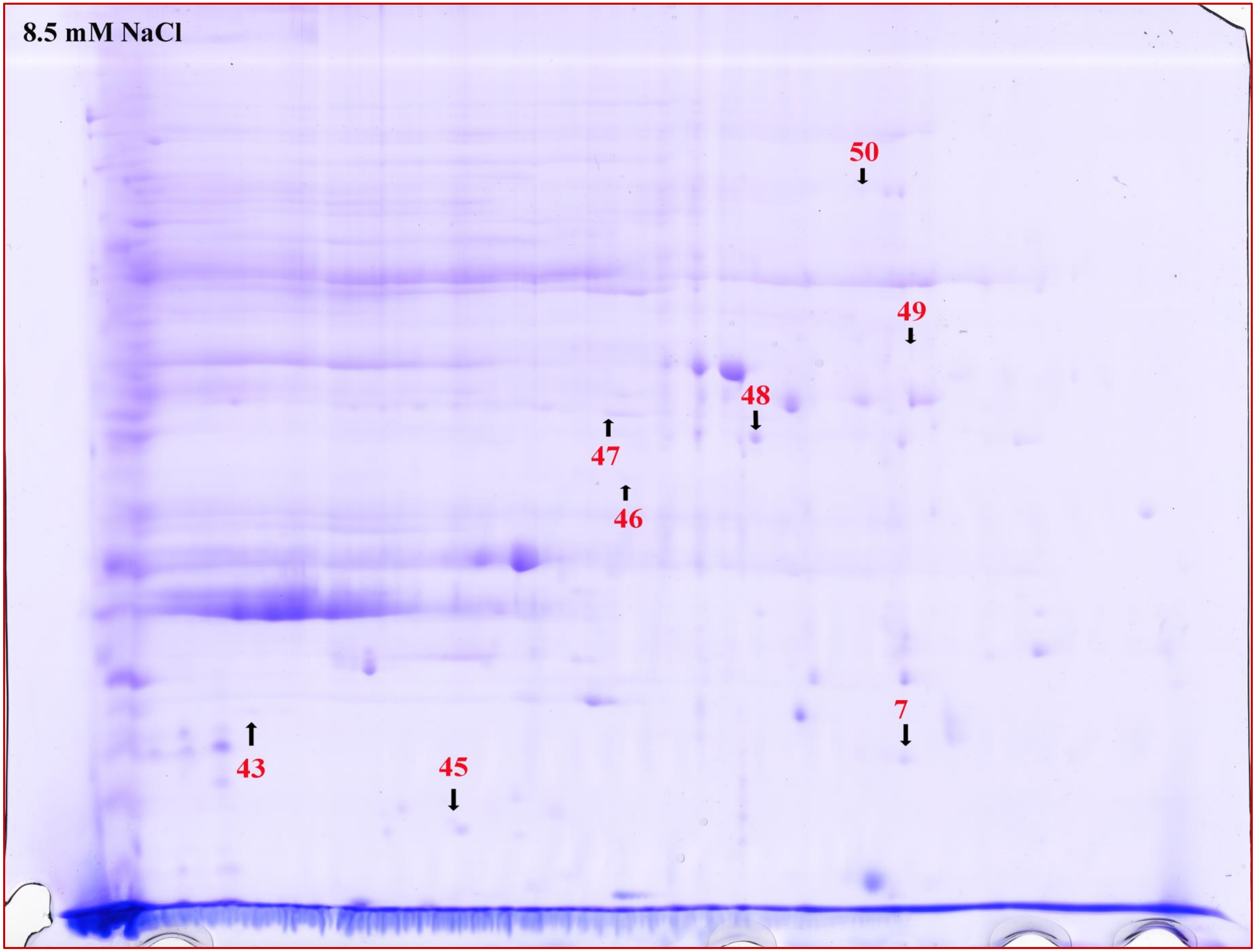

Fig. S6. A representative 2D-gel electrophoresis gel of *P. pringsheimii* total protein under **8.5 mM NaCl stress**. 550 µg of sample were loaded onto a 4-7 PI linear IPG strip and subject to isoelectric focusing (IEF) in the first dimension. The IPG strips were then loaded on a 12% SDS-PAGE gel for separation of proteins by size in the second dimension. Proteins were stained with Coomassie Blue.

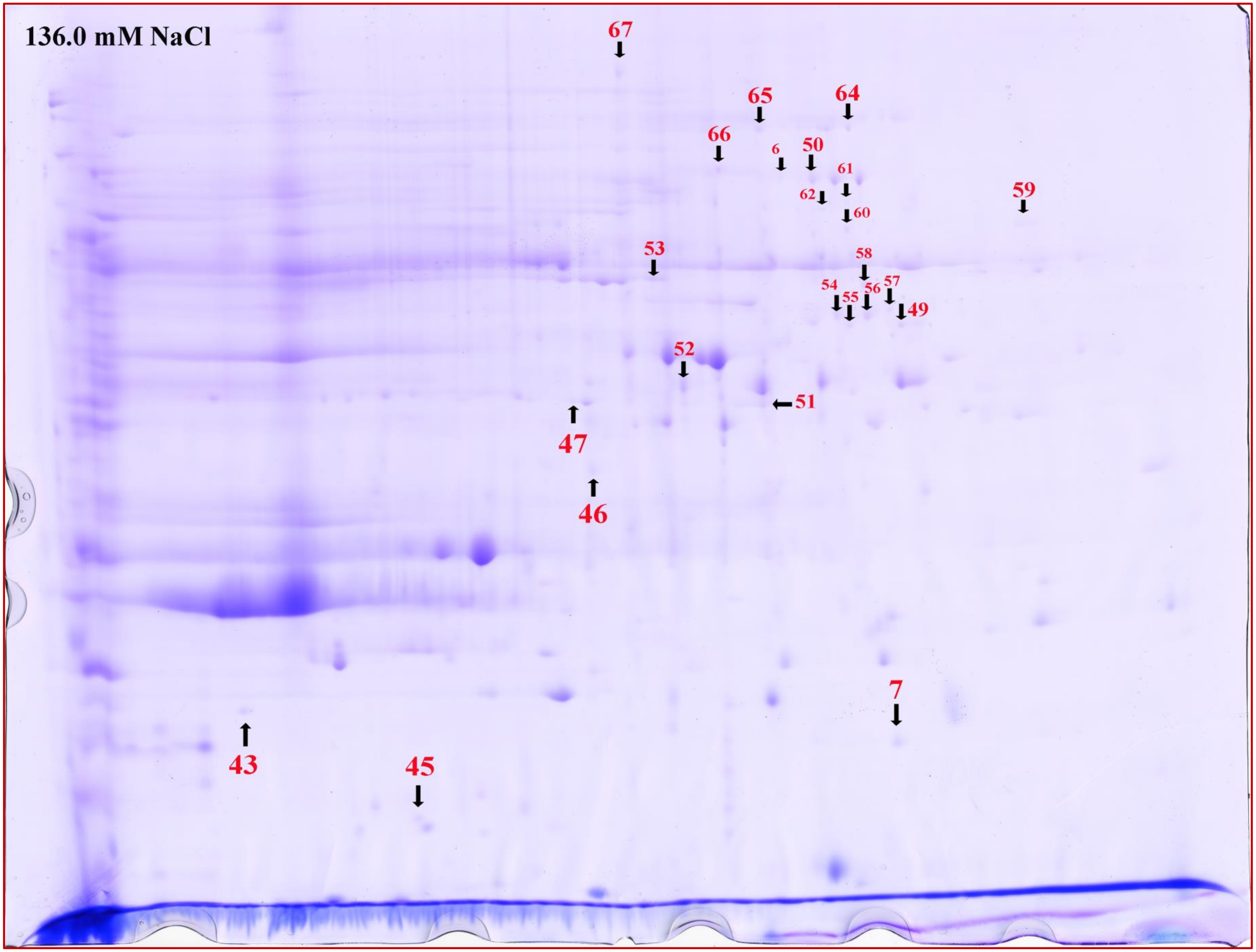

Fig. S7. A representative 2D-gel electrophoresis gel of *P. pringsheimii* total protein under **136.0 mM NaCl stress.** 550 µg of sample were loaded onto a 4-7 PI linear IPG strip and subject to isoelectric focusing (IEF) in the first dimension. The IPG strips were then loaded on a 12% SDS-PAGE gel for separation of proteins by size in the second dimension. Proteins were stained with Coomassie Blue.
