# Supplementary material for: Biochemical and proteomic response of the freshwater green alga Pseudochlorella pringsheimii to iron and salinity stressors
Source: BMC Plant Biol. 2024 Jan 10;24:42. doi: 10.1186/s12870-023-04688-9 (PMC10777535; doi:10.1186/s12870-023-04688-9)
Supplement: Supplementary file 2 — Supplementary Material 2 [file 12870_2023_4688_MOESM2_ESM.pdf]

Table S1. differently expressed peptides, and the name of protein class, identified by the gel-based approach of *P. pringsheimii* under the investigated stressors (0.35 mM Fe, and 136 mM NaCl).

Functional Class	#	Identified Proteins (77)	Accession Number	Molecular Weight (kDa)	Control	Iron	Salinity
Energy metabolism (ATP Synthesis)	2	ATP synthase CF1 beta subunit [*Scenedesmus obliquus*]	gi|108773085	52	14	13	16
	5	ATP synthase CF1 alpha subunit [*Scenedesmus obliquus*]	gi|108773075	55	8	9	10
	9	beta subunit of mitochondrial ATP synthase [*Chlamydomonas reinhardtii*]	gi|159466892	62	8	8	8
	16	Phosphoglycerate kinase [*Chlamydomonas reinhardtii*]	gi|1172455	49	4	4	4
	32	ATP synthase beta subunit [*Gastridium ventricosum*]	gi|110915642	53	2	0	2
	46	ATP synthase beta subunit [*Coriaria ruscifolia*]	gi|66276267	51	2	0	2
	53	ATP synthase CF1 beta chain [*Thalassiosira pseudonana*]	gi|118411134	51	0	2	2
	56	ATPase beta subunit [*Juniperus communis*]	gi|20467403	38	0	0	2
	70	ATP synthase beta-subunit [*Pediastrum duplex*]	gi|23503591	41	2	0	2
	71	chloroplast ATP synthase gamma chain [*Chlamydomonas reinhardtii*]	gi|159476472	39	0	0	2
	72	ATP synthase beta-subunit [*Volvox ovalis*]	gi|343430016	-	2	0	2
	76	ATP synthase beta subunit [*Stenomeris borneensis*]	gi|17224745	51	2	0	0
				**Sum:**	**44**	**36**	**52**
Carbohydrate metabolism	3	ribulose-1,5-bisphosphate carboxylase/oxygenase large subunit [*Stichococcus bacillaris*]	gi|119220502	43	8	6	9
	8	ribulose-1,5-bisphosphate carboxylase [*Spigelia anthelmia*]	gi|2961315	53	2	2	4
	24	sedoheptulose-1,7-biphosphatase [Volvox *carteri f. nagariensis*]	gi|302830724	-	2	2	2
	25	ribulose-1,5-bisphosphate carboxylase/oxygenase large subunit [*Lasiocroton bahamensis*]	gi|62003627	52	3	2	3
	29	ribulose 1,5-bisphosphate carboxylase [*Tribeles australis*]	gi|9910002	52	2	2	2
	38	Glyceraldehyde-3-phosphate dehydrogenase, gi|17978190| [*Scenedesmus vacuolatus*]	gi|75304410	34	2	2	4
	45	enolase [*Dunaliella salina*]	gi|29650775	52	0	2	0
	54	ribulose-1,5-bisphosphate carboxylase/oxygenase large subunit [*Parapediastrum biradiatum*]	gi|154423060	46	2	2	2
	60	fructose-1,6-bisphosphatase [*Volvox carteri f. nagariensis*]	gi|302833213	-	0	2	2
	69	ribulose-1,5-bisphosphate carboxylase/oxygenase large subunit [*Prostanthera rotundifolia*]	gi|1141727	48	2	0	2
	73	ribulose-1,5-bisphosphate carboxylase/oxygenase large subunit [*Erisma floribundum*]	gi|7240556	52	0	0	2
				**Sum:**	**23**	**22**	**32**
Regulation	1	Chain A, Trypsin In Complex With Borate, gi|110590763|pdb|2A32|	gi|110590762	23	4	4	4
	14	membrane AAA-metalloprotease [*Chlamydomonas reinhardtii*], gi|158279575|gb|EDP05335.1|	gi|159465357	78	6	6	4
	15	beta tubulin 2 [*Chlamydomonas reinhardtii*], gi|159471856|	gi|159471706	50	3	4	8
	17	actin [*Chlamydomonas reinhardtii*], gi|1703151|	gi|159482014	42	3	3	3
	20	chaperonin 60B2 [*Chlamydomonas reinhardtii*], gi|158278217|gb|EDP03982.1|	gi|159468684	62	3	4	2
	43	actin [*Chlorella vulgaris*]	gi|1519401	31	2	2	2
	51	alpha tubulin 1 [*Chlamydomonas reinhardtii*], gi|159490284| gi|158270806|gb|EDO96640.1| alpha tubulin 2 [*Chlamydomonas reinhardtii*], gi|158278603|gb|EDP04366.1|	gi|159467393	50	0	0	5
	59	S-Adenosyl homocysteine hydrolase [*Chlamydomonas reinhardtii*], gi|158277597|gb|EDP03365.1|	gi|159470383	53	2	0	2
				**Sum:**	**23**	**23**	**30**

**Photosynthesis**	**6**	light-harvesting chlorophyll-a/b binding protein LhcbM2 [*Scenedesmus obliquus*]	gi|87313205	26	2	0	3
	**7**	33kDa oxygen evolving protein of photosystem II [*Hafniomonas montana*]	gi|161728799	31	2	2	0
	**12**	oxygen-evolving enhancer protein [*Scenedesmus obliquus*]	gi|288816167	-	2	3	0
	**19**	photosystem II 44 kDa protein [*Scenedesmus obliquus*], gi|122179541|sp|Q1KVY2.1|	gi|108773032	51	3	3	4
	**23**	photosystem II protein D2 [*Scenedesmus obliquus*], gi|122237658|sp|Q1KVW6.1|	gi|108773048	40	3	4	4
	**26**	photosystem I subunit VII [*Volvox carteri*], gi|261888149|gb|ACY06004.1|	gi|208751286	9	4	3	3
	**31**	chlorophyll apoprotein of photosystem II 47 kDa protein [*Scenedesmus obliquus*], gi|88696625|gb|ABD48251.1|	gi|108773059	56	3	0	5
	**37**	PSII D1 protein [*Prunus cerasoides var. campanulata*]	gi|15529745	39	2	0	2
	**65**	cytochrome f [*Dunaliella salina*]	gi|246880725	31	0	2	0
				**Sum:**	**21**	**17**	**21**
**Protein synthesis**	**11**	eukaryotic translation elongation factor 1 alpha 1 [*Chlamydomonas reinhardtii*], gi|159488713|	gi|159476938	51	7	5	6
	**30**	elongation factor Tu [*Scenedesmus obliquus*], gi|122225248|sp|Q1KVS9.1|, gi|88696652|gb|ABD48278.1|	gi|108773086	46	3	4	3
	**34**	translational elongation factor Tu [*Chlamydomonas moewusii*]	gi|156619327	46	2	2	3
	**41**	eukaryotic initiation factor 4A (ATP-dependent RNA helicase eIF4A) [*Chlorella variabilis*]	gi|307104817	-	4	0	2
				**Sum:**	**16**	**11**	**14**
**Stress proteins**	**13**	heat shock protein 70A [*Chlamydomonas reinhardtii*], gi|158271809|gb|EDO97621.1|	gi|159486599	71	5	6	5
	**35**	luminal binding protein Bip1 [*Volvox carteri f. nagariensis*]	gi|302836031	-	3	3	3
	**40**	peroxiredoxin [*Chlamydomonas reinhardtii*]	gi|11995220	22	2	0	0
	**42**	thioredoxin/transketolase fusion protein [synthetic construct]	gi|25067747	87	3	0	0
	**52**	heat shock protein 90-2 [*Glycine max*]	gi|208964722	80	3	0	3
				**Sum:**	**16**	**9**	**11**
**Oxido-reductase R.**	**33**	enoyl-ACP reductase [*Polytomella parva*]	gi|111608895	36	2	2	0
	**44**	malate dehydrogenase [*Micromonas* sp. RCC299], gi|226515895|gb|ACO61890.1|	gi|255073915	33	0	2	2
	**47**	malate dehydrogenase precursor [*Medicago sativa*]	gi|2827084	43	2	2	2
	**55**	malate dehydrogenase [*Volvox carteri f. nagariensis*]	gi|302846584	-	2	2	0
	**57**	putative short-chain dehydrogenase [*Deinococcus deserti* VCD115], gi|226319905|gb|ACO47899.1	gi|226358262	32	0	0	2
	**63**	NADP-Malate dehydrogenase [*Chlamydomonas reinhardtii*], gi|19069739|emb|CAC19083.2|	gi|15947737	45	2	2	2
				**Sum:**	**8**	**10**	**8**
**Transfer proteins**	**28**	phosphoribulokinase [*Chlamydomonas reinhardtii*], gi|125577|sp|P19824.1|, gi|7107404|gb|AAF36402.1|, gi|167432|gb|AAA33090.1|, gi|158277205|gb|EDP02974.1|, gi|227431|	gi|159471788	42	2	2	3
	**64**	aspartate aminotransferase [*Chlamydomonas reinhardtii*], gi|158281981|gb|EDP07735.1|	gi|159483981	47	2	2	2
	**74**	dihydrolipoamide acetyltransferase [*Chlamydomonas reinhardtii*], gi|158274386|gb|EDP00169.1|	gi|159478837	43	0	2	0
				**Sum:**	**4**	**6**	**5**
**Ribonucleic -associated proteins**	**61**	plastid ribosomal protein L1 [*Chlamydomonas reinhardtii*], gi|158280980|gb|EDP06736.1|	gi|159487501	32	2	0	0
	**68**	ATP-dependent RNA helicase [*Oryza sativa* (japonica cultivar-group)], gi|75326432|, gi|41469394|gb|AAS07217.1|	gi|115456051	68	0	2	0
				**Sum:**	**2**	**2**	**0**
**Hypothetical proteins**	**18**	hypothetical protein VOLCADRAFT_121148 [*Volvox carteri f. nagariensis*]	gi|302843549	-	3	2	3
	**21**	hypothetical protein VOLCADRAFT_105311 [*Volvox carteri f. nagariensis*]	gi|302840740	-	3	4	0
	**22**	hypothetical protein VOLCADRAFT_79395 [*Volvox carteri f. nagariensis*]	gi|302830850	-	2	3	2
	**27**	hypothetical protein SELMODRAFT_167539 [*Selaginella moellendorffii*]	gi|302764040	-	2	2	2
	**36**	hypothetical protein VOLCADRAFT_109207 [*Volvox carteri f. nagariensis*]	gi|302843948	-	3	4	3
	**39**	hypothetical protein VOLCADRAFT_75893 [*Volvox carteri f. nagariensis*]	gi|302844301	-	2	2	2

	**48**	hypothetical protein VOLCADRAFT_78972 [*Volvox carteri f. nagariensis*]	gi|302829468	-	0	2	3
	**50**	hypothetical protein CHLNCDRAFT_18230 [*Chlorella variabilis*]	gi|307111876	-	2	2	3
	**62**	hypothetical protein VOLCADRAFT_77870 [*Volvox carteri f. nagariensis*]	gi|302853472	-	0	0	2
	**66**	hypothetical protein VOLCADRAFT_89892 [*Volvox carteri f. nagariensis*]	gi|302835754	-	2	2	0
	**18**	hypothetical protein VOLCADRAFT_121148 [*Volvox carteri f. nagariensis*]	gi|302843549	-	3	2	3
				**Sum:**	**19**	**23**	**20**
**Other proteins**	**4**	predicted protein [*Physcomitrella patens* subsp. patens], gi|162693081|gb|EDQ79435.1|	gi|168006129	28	2	3	3
	**10**	predicted protein [*Physcomitrella patens* subsp. patens], gi|162690324|gb|EDQ76692.1|	gi|168012047	42	3	3	3
	**49**	predicted protein [*Micromonas pusilla* CCMP1545]	gi|303272845	-	2	2	2
	**75**	14-3-3f protein [*Gossypium hirsutum*]	gi|302122826	-	2	0	0
	**77**	gi|299560687	gi|299560687	-	2	0	0
				**Sum:**	**11**	**8**	**8**
**Unknown**	**58**	unknown [*Populus trichocarpa*]	gi|118481419	16	2	0	0
	**67**	unknown [*Glycine max*]	gi|255634854	36	0	0	2
				**Sum:**	**2**	**0**	**2**
				**Total: 559**	**189**	**167**	**203**
